# Prevalence, functional impact, and associated factors of knee osteoarthritis and knee pain among adults in Urumqi, Northwest China: a community-based cross-sectional study

**DOI:** 10.3389/fpubh.2026.1903768

**Published:** 2026-09-11

**Authors:** Lingyun Shi, Siqi Li, Jiaju Zhao, Guoliang Hou, Abiding Abulaiti, Xiaomin Lin, Haitao Zhang, Palida Maimaiti

**Affiliations:** 1 School of Nursing, Xinjiang Medical University, Urumqi, China; 2 The First Affiliated Hospital of Xinjiang Medical University, Urumqi, China

**Keywords:** community-based survey, functional impact, knee osteoarthritis, knee pain, modifiable risk factors

## Abstract

**Background:**

Knee osteoarthritis and knee pain are major causes of chronic pain, functional limitation, and health-care use, but community-level evidence on their symptomatic burden and associated factors remains limited in northwest China. We aimed to estimate the burden of knee osteoarthritis and knee pain, describe pain-related functional impact, and examine demographic, clinical, lifestyle, and occupational factors associated with these outcomes among adults in Urumqi.

**Methods:**

We conducted a community-based cross-sectional survey in Urumqi, Xinjiang, China, from June to August 2023. The primary analysis included adults aged 40 years or older with valid anthropometric and core knee outcome data. Outcomes were self-reported physician-diagnosed knee osteoarthritis, past-year knee pain, symptomatic knee osteoarthritis, and pain-related functional impact. Modified Poisson regression with robust variance was used to estimate adjusted prevalence ratios (aPRs) and 95% CIs.

**Results:**

Among 1925 participants, the median age was 55.0 years (IQR 48.0–63.0), and 1,118 (58.1%) were men. Physician-diagnosed knee osteoarthritis was reported by 236 participants (12.3, 95% CI 10.9–13.8), past-year knee pain by 562 (29.2%, 27.2–31.3), and symptomatic knee osteoarthritis by 212 (11.0%, 9.7–12.5). Among participants with past-year knee pain, 214 of 525 reported sleep disturbance (40.8%, 36.6–45.0), 286 of 525 reported work or activity limitation (54.5%, 50.2–58.7), and 320 of 525 had sought medical care (61.0%, 56.7–65.0). Previous knee injury was strongly associated with physician-diagnosed knee osteoarthritis (aPR 3.08, 95% CI 2.37–4.00), past-year knee pain (2.01, 1.72–2.34), and symptomatic knee osteoarthritis (2.97, 2.25–3.91). Sleep disorder, overweight, obesity, high occupational mechanical load, and first-degree family history of arthritis were also associated with knee outcomes.

**Conclusion:**

Knee osteoarthritis and knee pain were common among middle-aged and older adults in Urumqi and were frequently accompanied by sleep disturbance, activity limitation, and health-care seeking. The observed associations with previous knee injury, excess body weight, occupational mechanical load, and sleep disorder identify groups that may warrant closer community assessment; their temporal and causal roles require confirmation in longitudinal studies.

## 1 Introduction

Knee osteoarthritis (KOA) is a major cause of chronic pain and disability, and its burden is increasing with population ageing and rising adiposity. The knee is the most commonly affected joint, and global analyses show sustained increases in prevalence and years lived with disability, particularly among women and older adults (1–4).

The public health burden of KOA is not captured by structural disease or diagnostic history alone. Knee pain and its effects on mobility, sleep, work, and health-care use often determine the need for care and the extent of disability (5, 6). Community-level data that jointly describe physician-diagnosed KOA, recent knee pain, and pain-related functional consequences are therefore important for local service planning.

National surveys and modelling studies describe the scale and distribution of KOA, but provide less information on how diagnostic history and symptoms coexist with functional limitation and locally relevant occupational, anthropometric, sleep, injury, and comorbidity characteristics. Such evidence remains limited in northwest China, where population ageing, work patterns, digital access, and routes to health care may differ from those in more extensively studied regions.

We therefore conducted a community-based cross-sectional survey in Urumqi, Xinjiang, to estimate the survey-based burden of self-reported physician-diagnosed KOA, past-year knee pain, and symptomatic KOA; describe pain-related functional impact and health-care seeking; and examine demographic, clinical, lifestyle, and occupational factors associated with these outcomes. The study was designed to provide locally relevant descriptive and associational evidence, rather than to establish causal effects.

## 2 Methods

### 2.1 Study design and participants

**Figure 1:**
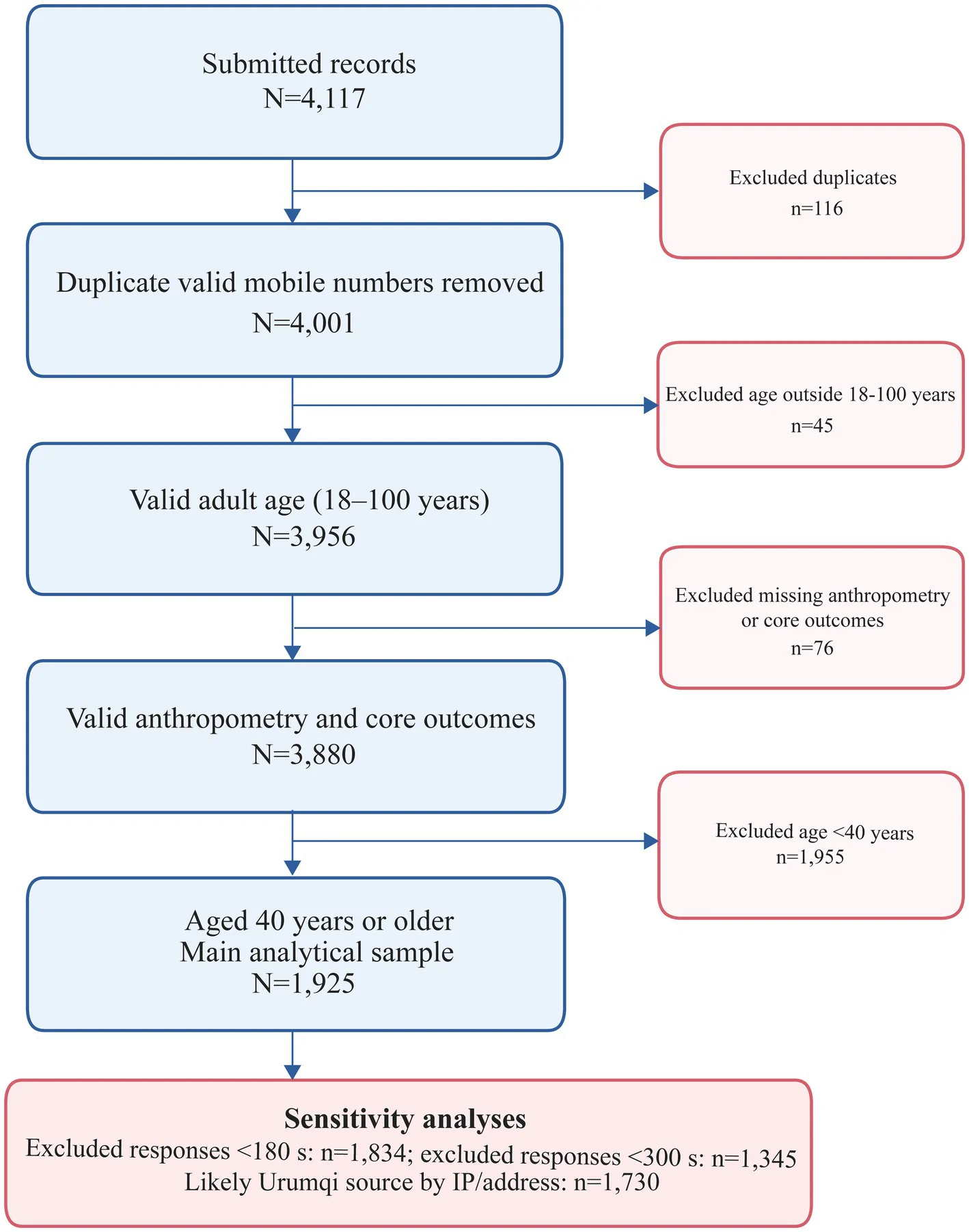
Study population and analytical sample construction. Among the 76 participants excluded after age screening, mutually exclusive sequential reasons were invalid or missing height (*n* = 25), invalid or missing weight among those with valid height (*n* = 9), and BMI outside 15–45 kg/m^2^ despite valid height and weight (*n* = 42). No participant was excluded for missing physician-diagnosed KOA or past-year knee-pain status.

We conducted a cross-sectional, community-based survey in Urumqi City, Xinjiang, China, from June to August 2023. Adults aged 18 years or older who had resided in the city for at least six months were eligible. Participants completed an electronic questionnaire distributed via WeChat and community outreach. Each record was linked to a unique mobile phone number to prevent duplicate submissions. Records with duplicate numbers, ages outside 18–100 years, or missing core outcome data were excluded (Figure 1).

The study protocol was approved by the Ethics Committee of the First Affiliated Hospital of Xinjiang Medical University (approval number 230306-112). The present survey constituted the cross-sectional epidemiological assessment component of a broader ethics-approved project on KOA management; the submitted ethics materials included the community questionnaire, recruitment procedures, and consent process. No intervention or follow-up treatment data were used in the present analysis. All participants provided written electronic informed consent, and data were anonymised before analysis. The complete questionnaire is provided as Supplementary File S1, and reporting follows the Strengthening the Reporting of Observational Studies in Epidemiology (STROBE) statement (7).

### 2.2 Knee osteoarthritis and knee pain assessment

Primary outcomes were self-reported physician-diagnosed knee osteoarthritis and past-year knee pain. Physician-diagnosed knee osteoarthritis was defined by an affirmative response to the questionnaire item, “Have you ever been diagnosed with knee osteoarthritis by a doctor?” Past-year knee pain was defined by an affirmative response to “Have you had knee pain during the past 12 months?”; the questionnaire did not require a minimum frequency, duration, or severity threshold. Symptomatic knee osteoarthritis was defined as the coexistence of these two self-reported outcomes. Among participants reporting a history of knee pain, pain-related functional impact was assessed directly with three binary items asking whether knee pain affected sleep, affected work or other activities, or prompted medical help-seeking. Two further study-specific binary items assessed knee pain after exercise and in cold weather during the past 12 months. Because these outcomes were not confirmed by imaging, clinical examination, or medical records, they were treated as survey-based measures rather than clinically verified diagnoses (8).

### 2.3 Covariates

Self-reported sociodemographic variables included age, sex, ethnicity, residence, education, occupation, and monthly income. Monthly income referred to the participant’s own self-reported monthly income in Chinese yuan and was grouped as <3,000, 3,000–4,999, 5,000–7,999, and ≥8,000 CNY. Self-reported height and weight were used to calculate BMI (kg/m^2^). BMI was categorised according to the Working Group on Obesity in China criteria as underweight (<18.5 kg/m^2^), normal weight (18.5–23.9 kg/m^2^), overweight (24.0–27.9 kg/m^2^), or obesity (≥28.0 kg/m^2^) (9). Smoking status, weekly exercise, and routine regularity were obtained from separate questionnaire items.

Occupational mechanical load was represented by a study-specific score based on four current-work items: prolonged standing or walking, frequent knee bending or twisting, repeated squatting or stair climbing, and handling heavy loads. Each affirmative response was assigned 1 point and each negative response 0 points; item scores were summed to give a total from 0 to 4. Scores were analysed as 0, 1, 2, and 3–4, with the two upper categories combined to avoid sparse cells. This score was not a previously validated instrument and reflected concurrent work requirements rather than cumulative lifetime exposure. Sleep disorder and irregular routine were assessed as separate binary self-reports using the questions “Do you have a sleep disorder?” and “Is your daily routine regular?”, respectively. The metabolic comorbidity count comprised self-reported hypertension, diabetes, hyperlipidaemia, and hyperuricaemia and was categorised as 0, 1, or ≥2 conditions. Previous knee injury was self-reported. First-degree family history of arthritis was defined as any reported arthritis in a participant’s father, mother, or full sibling.

### 2.4 Statistical analysis

Analyses were conducted using R (version 4.3.2). Inspection of histograms and quantile plots indicated that age and BMI were not normally distributed; continuous variables were therefore summarised as medians with IQRs and compared with Wilcoxon rank-sum tests. Categorical variables were summarised as counts and percentages and compared with chi-square tests. Prevalence estimates were accompanied by Wilson score 95% CIs because these intervals have more reliable coverage than normal-approximation intervals, particularly for subgroup estimates with smaller denominators (10).

To obtain directly interpretable prevalence ratios, modified Poisson regression with robust error variance was selected *a priori* for the binary outcomes. This approach avoids the potential overstatement associated with odds ratios for common outcomes and is less prone to boundary-related convergence problems than log-binomial regression (11, 12). Exploratory log-binomial models using the same adjustment sets were attempted, but did not converge reliably for all three outcomes; modified Poisson models were therefore retained. The main factors of interest were BMI category, sleep disorder, occupational mechanical load score, previous knee injury, metabolic comorbidity count, and first-degree family history of arthritis. Models additionally adjusted *a priori* for age, sex, ethnicity, residence, education, monthly income, smoking status, weekly exercise, and routine regularity on the basis of epidemiological relevance rather than univariable statistical significance. The same adjustment set was used for physician-diagnosed KOA, past-year knee pain, and symptomatic KOA. BMI, metabolic comorbidity, and sleep disorder were retained because they represented distinct anthropometric, cardiometabolic, and symptom domains; potential multicollinearity was assessed using variance inflation factors. The three primary models included 26 covariate parameters and 236, 562, and 212 outcome events, respectively, corresponding to 9.1, 21.6, and 8.2 events per parameter. These ratios were used descriptively rather than as fixed adequacy thresholds; all models converged, and estimates for symptomatic KOA were interpreted with particular attention to CI width (13).

For participants reporting past-year knee pain, functional-impact outcomes were examined in secondary, exploratory modified Poisson models using a prespecified parsimonious set of age, sex, BMI category, sleep disorder, occupational load score 3–4, previous knee injury, metabolic comorbidity, and first-degree family history of arthritis. Primary estimates are presented with unadjusted two-sided *p* values because the exposure–outcome comparisons were prespecified; however, a Benjamini–Hochberg false-discovery-rate sensitivity analysis was applied across the 45 prespecified exposure–outcome comparisons, and the smaller functional-impact analyses were interpreted as exploratory (14). Sensitivity analyses also excluded rapid responses (<180 s and <300 s), restricted the sample to participants with likely Urumqi residency according to IP address or residential address, excluded records with logical conflicts, repeated the analyses among all adults aged 18 years or older, and restricted the primary population to adults aged 45 years or older.

All variables entered into the primary regression models were complete in the main analytical sample; therefore, the primary analyses used complete-case data, no multiple imputation was performed, and a complete-case versus multiple-imputation comparison was not applicable. Conditional pain-impact items had item-specific denominators because of questionnaire skip logic and item nonresponse and were analysed among participants with non-missing responses to the corresponding item. All tests were two-sided. Effect estimates and 95% CIs were prioritised over dichotomous interpretation of the conventional *p* < 0.05 threshold.

## 3 Results

### 3.1 Study population

A total of 4,117 questionnaire records were submitted. After exclusion of 116 duplicate records identified from valid mobile phone numbers, 4,001 records remained. Of these, 45 were excluded because age was outside 18–100 years. Among the remaining 3,956 participants, 76 were excluded using mutually exclusive sequential criteria: 25 had invalid or missing height, nine had invalid or missing weight after removal of those with invalid height, and 42 had a calculated BMI outside 15–45 kg/m^2^ despite valid height and weight. No participant was excluded because physician-diagnosed KOA or past-year knee-pain status was missing. The resulting 3,880 participants had valid anthropometric information and core outcomes; 1925 adults aged 40 years or older formed the primary analytical sample. Prespecified sensitivity analyses excluded responses completed in less than 180 s (*n* = 1834), excluded responses completed in less than 300 s (*n* = 1,345), and restricted the sample to a likely Urumqi source according to IP address or residential address (*n* = 1730; Figure 1; Supplementary Table S1). No values were missing for variables included in the primary regression models; conditional pain-impact items had item-specific denominators because of skip logic and item nonresponse.

### 3.2 Baseline characteristics

Among the 1925 participants included in the main analysis, the median age was 55.0 years (IQR 48.0–63.0), and 1,118 (58.1%) were men. Overall, 1,406 (73.0%) participants were Han Chinese and 519 (27.0%) were classified as other ethnic groups. Most participants lived in urban areas (1,655 [86.0%]). The median BMI was 23.9 kg/m^2^ (IQR 21.6–26.4); 696 (36.2%) participants were overweight and 258 (13.4%) had obesity. Sleep disorder was reported by 454 (23.6%) participants, 423 (22.0%) had a high occupational mechanical load score of 3–4, 184 (9.6%) reported previous knee injury, and 342 (17.8%) reported a first-degree family history of arthritis (Table 1).

**Table 1:** Characteristics of participants aged 40 years or older, by self-reported physician-diagnosed knee osteoarthritis.

Characteristic	Overall (*n* = 1925)	No KOA (*n* = 1,689)	KOA (*n* = 236)	*p* value
Age, years	55.0 (48.0–63.0)	55.0 (48.0–62.0)	58.0 (49.0–65.0)	0.006
Age group				
40–49	621 (32.3%)	553 (32.7%)	68 (28.8%)	0.232
50–59	651 (33.8%)	577 (34.2%)	74 (31.4%)	
60–69	416 (21.6%)	357 (21.1%)	59 (25.0%)	
≥70	237 (12.3%)	202 (12.0%)	35 (14.8%)	
Sex				
Male	1,118 (58.1%)	1014 (60.0%)	104 (44.1%)	<0.001
Female	807 (41.9%)	675 (40.0%)	132 (55.9%)	
Ethnicity				
Han	1,406 (73.0%)	1250 (74.0%)	156 (66.1%)	0.013
Other	519 (27.0%)	439 (26.0%)	80 (33.9%)	
Residence				
Urban	1,655 (86.0%)	1456 (86.2%)	199 (84.3%)	0.316
Suburban	21 (1.1%)	20 (1.2%)	1 (0.4%)	
Rural	249 (12.9%)	213 (12.6%)	36 (15.3%)	
Education				
Primary or below	307 (15.9%)	258 (15.3%)	49 (20.8%)	0.004
Junior/senior high	913 (47.4%)	824 (48.8%)	89 (37.7%)	
College or above	705 (36.6%)	607 (35.9%)	98 (41.5%)	
Monthly income, CNY				
<3,000	425 (22.1%)	367 (21.7%)	58 (24.6%)	0.350
3,000–4,999	598 (31.1%)	520 (30.8%)	78 (33.1%)	
5,000–7,999	645 (33.5%)	578 (34.2%)	67 (28.4%)	
≥8,000	257 (13.4%)	224 (13.3%)	33 (14.0%)	
Occupation				
Manual/physical	263 (13.7%)	223 (13.2%)	40 (16.9%)	0.054
Office/professional/student	830 (43.1%)	716 (42.4%)	114 (48.3%)	
Other/unknown	568 (29.5%)	509 (30.1%)	59 (25.0%)	
Self-employed/flexible	203 (10.5%)	187 (11.1%)	16 (6.8%)	<0.001
Unemployed	61 (3.2%)	54 (3.2%)	7 (3.0%)	
BMI, kg/m^2^	23.9 (21.6–26.4)	23.7 (21.5–26.3)	25.0 (22.9–27.0)	
BMI category				
Underweight	56 (2.9%)	55 (3.3%)	1 (0.4%)	<0.001
Normal	915 (47.5%)	827 (49.0%)	88 (37.3%)	
Overweight	696 (36.2%)	592 (35.1%)	104 (44.1%)	
Obesity	258 (13.4%)	215 (12.7%)	43 (18.2%)	
Smoking status				
Never	1234 (64.1%)	1061 (62.8%)	173 (73.3%)	0.006
Former	315 (16.4%)	284 (16.8%)	31 (13.1%)	
Current	376 (19.5%)	344 (20.4%)	32 (13.6%)	
Weekly exercise				
≥2.5 h/week	281 (14.6%)	257 (15.2%)	24 (10.2%)	0.007
1–2.5 h/week	844 (43.8%)	751 (44.5%)	93 (39.4%)	
≤1 h/week	800 (41.6%)	681 (40.3%)	119 (50.4%)	
Sleep disorder				
Yes	454 (23.6%)	337 (20.0%)	117 (49.6%)	<0.001
No	1471 (76.4%)	1352 (80.0%)	119 (50.4%)	
Irregular routine				
Yes	617 (32.1%)	557 (33.0%)	60 (25.4%)	0.024
No	1308 (67.9%)	1132 (67.0%)	176 (74.6%)	
Occupational load score				
0	1153 (59.9%)	1043 (61.8%)	110 (46.6%)	<0.001
1	239 (12.4%)	202 (12.0%)	37 (15.7%)	
2	110 (5.7%)	94 (5.6%)	16 (6.8%)	
3–4	423 (22.0%)	350 (20.7%)	73 (30.9%)	
Prior knee injury				
Yes	184 (9.6%)	100 (5.9%)	84 (35.6%)	<0.001
No	1741 (90.4%)	1589 (94.1%)	152 (64.4%)	
Metabolic comorbidity count				
0	966 (50.2%)	863 (51.1%)	103 (43.6%)	0.001
1	677 (35.2%)	597 (35.3%)	80 (33.9%)	
≥2	282 (14.6%)	229 (13.6%)	53 (22.5%)	
First-degree family history of arthritis				
Yes	342 (17.8%)	253 (15.0%)	89 (37.7%)	<0.001
No	1583 (82.2%)	1436 (85.0%)	147 (62.3%)	

Data are median (IQR) or *n* (%). Age and BMI were non-normally distributed and were compared with Wilcoxon rank-sum tests; categorical variables were compared with chi-square tests. No values were missing for the variables shown, and This table presents the original complete-case data. KOA = knee osteoarthritis.

Participants with self-reported physician-diagnosed knee osteoarthritis differed from those without knee osteoarthritis in several clinically relevant characteristics. They were older (median age 58.0 years [IQR 49.0–65.0] vs. 55.0 years [48.0–62.0]; *p* = 0.006), more often female (132 [55.9%] of 236 vs. 675 [40.0%] of 1,689; *p* < 0.001), and had a higher median BMI (25.0 kg/m^2^ [IQR 22.9–27.0] vs. 23.7 kg/m^2^ [21.5–26.3]; *p* < 0.001). They were also more likely to report sleep disorder (117 [49.6%] vs. 337 [20.0%]), previous knee injury (84 [35.6%] vs. 100 [5.9%]), two or more metabolic comorbidities (53 [22.5%] vs. 229 [13.6%]), and a first-degree family history of arthritis (89 [37.7%] vs. 253 [15.0%]; all *p* < 0.001; Table 1).

### 3.3 Prevalence of knee osteoarthritis and knee pain

In the primary analytical sample, 236 participants reported physician-diagnosed knee osteoarthritis, corresponding to a prevalence of 12.3% (95% CI 10.9–13.8). Past-year knee pain was reported by 562 participants (29.2, 95% CI 27.2–31.3). Symptomatic knee osteoarthritis, defined as physician-diagnosed knee osteoarthritis with concurrent past-year knee pain, was present in 212 participants (11.0%, 9.7–12.5; Table 2).

**Table 2:** Burden of knee osteoarthritis, knee pain, and pain-related functional impact.

Outcome	Denominator	n/N	Prevalence (95% CI)
Self-reported physician-diagnosed knee osteoarthritis	Main sample (*n* = 1925)	236/1925	12.3% (10.9–13.8)
Past-year knee pain	Main sample (*n* = 1925)	562/1925	29.2% (27.2–31.3)
Symptomatic knee osteoarthritis (diagnosed KOA + past-year knee pain)	Main sample (*n* = 1925)	212/1925	11.0% (9.7–12.5)
Knee pain affecting sleep	Participants with past-year knee pain (*n* = 562)	214/525	40.8% (36.6–45.0)
Knee pain affecting work or activities	Participants with past-year knee pain (*n* = 562)	286/525	54.5% (50.2–58.7)
Sought medical help for knee pain	Participants with past-year knee pain (*n* = 562)	320/525	61.0% (56.7–65.0)
Exercise-related knee pain in past year	Participants with past-year knee pain (*n* = 562)	363/385	94.3% (91.5–96.2)
Cold-weather knee pain in past year	Participants with past-year knee pain (*n* = 562)	332/345	96.2% (93.7–97.8)
Any sleep or work/activity impact among those with past-year knee pain	Participants with past-year knee pain (*n* = 562)	309/562	55.0% (50.8–59.0)

Prevalence estimates are shown with Wilson 95% CIs. Of 562 participants with past-year knee pain, 37 reported no previous history of knee pain and therefore did not receive the conditional sleep, work/activity, and medical-help items, leaving *n* = 525. The exercise-related pain item was conditional on a history of exercise-related knee pain (*n* = 385), and the cold-weather item was conditional on a history of cold-weather knee pain (*n* = 345). The n/N values therefore reflect questionnaire skip logic and item-specific nonresponse rather than longitudinal follow-up.

**Figure 2:**
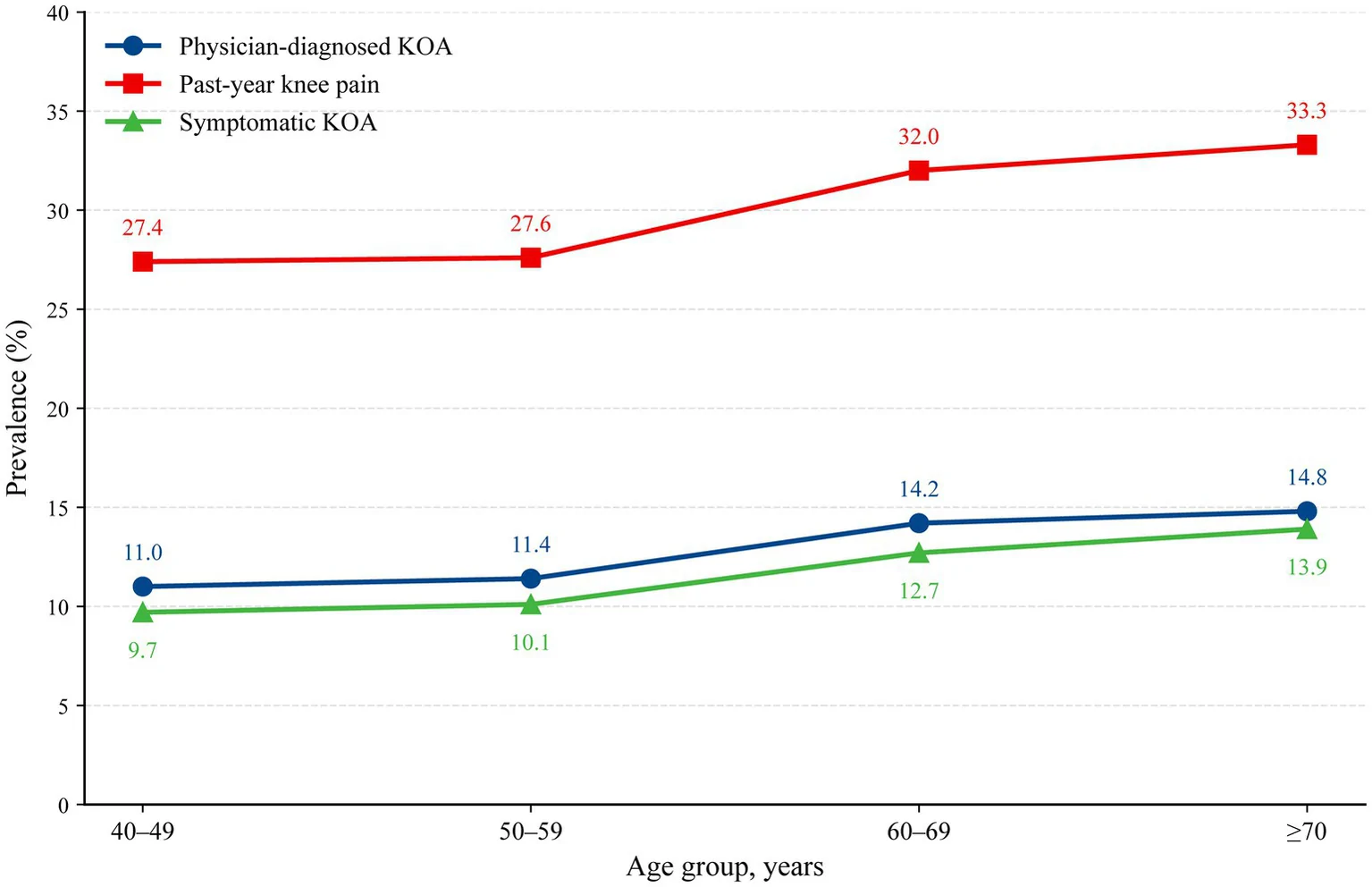
Age-specific prevalence of self-reported physician-diagnosed knee osteoarthritis, past-year knee pain, and symptomatic knee osteoarthritis.

The burden of all three outcomes rose with age. The prevalence of physician-diagnosed knee osteoarthritis increased from 11.0% (95% CI 8.7–13.7) among participants aged 40–49 years to 14.8% (10.8–19.8) among those aged 70 years or older. Over the same age range, past-year knee pain increased from 27.4% (24.0–31.0) to 33.3% (27.6–39.6), and symptomatic knee osteoarthritis increased from 9.7% (7.6–12.2) to 13.9% (10.1–18.9; Figure 2; Supplementary Table S2).

Women had a consistently higher burden than men. Past-year knee pain was reported by 342 (42.3, 95% CI 38.9–45.7) of 807 women and 221 (19.8%, 17.5–22.2) of 1,118 men. Physician-diagnosed knee osteoarthritis was also more common among women than men (16.4% [14.0–19.1] vs. 9.3% [7.7–11.1]), as was symptomatic knee osteoarthritis (15.1% [12.8–17.8] vs. 8.1% [6.6–9.8]; Supplementary Table S2 and Supplementary Figure S1).

### 3.4 Functional impact and health-care use among participants with past-year knee pain

**Figure 3:**
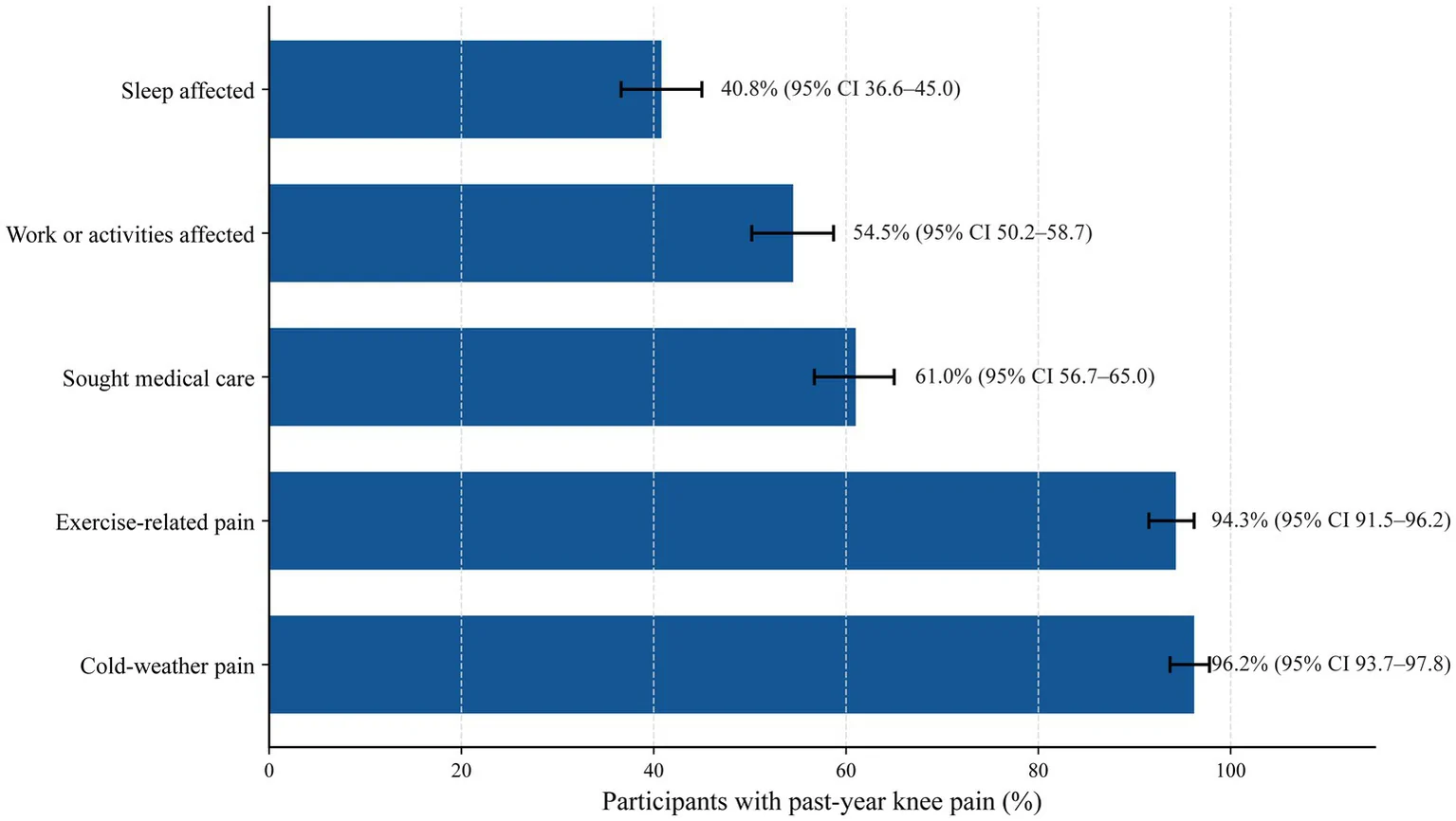
Pain-related functional impact and health-care seeking among participants with past-year knee pain.

Past-year knee pain was frequently accompanied by sleep disturbance, activity limitation, and health-care use. Among participants with past-year knee pain and non-missing responses to the corresponding conditional questionnaire items, 214 of 525 reported that knee pain affected sleep (40.8, 95% CI 36.6–45.0), 286 of 525 reported that knee pain affected work or daily activities (54.5%, 50.2–58.7), and 320 of 525 had sought medical care for knee pain (61.0%, 56.7–65.0). Among participants who completed the relevant conditional items, 363 of 385 reported exercise-related knee pain in the past year (94.3%, 91.5–96.2), and 332 of 345 reported cold-weather knee pain (96.2%, 93.7–97.8). Overall, 309 of 562 participants with past-year knee pain reported either sleep disturbance or work/activity limitation attributable to knee pain (55.0%, 50.8–59.0; Table 2; Figure 3).

### 3.5 Burden by BMI and occupational mechanical load

Knee outcomes varied across BMI categories. Physician-diagnosed knee osteoarthritis was reported by 88 (9.6, 95% CI 7.9–11.7) of 915 participants with normal BMI, 104 (14.9%, 12.5–17.8) of 696 participants with overweight, and 43 (16.7%, 12.6–21.7) of 258 participants with obesity. A similar pattern was seen for symptomatic knee osteoarthritis, which increased from 8.4% (6.8–10.4) in participants with normal BMI to 13.6% (11.3–16.4) in those with overweight and 15.5% (11.6–20.4) in those with obesity (Supplementary Table S2; Supplementary Figure S2).

**Figure 4:**
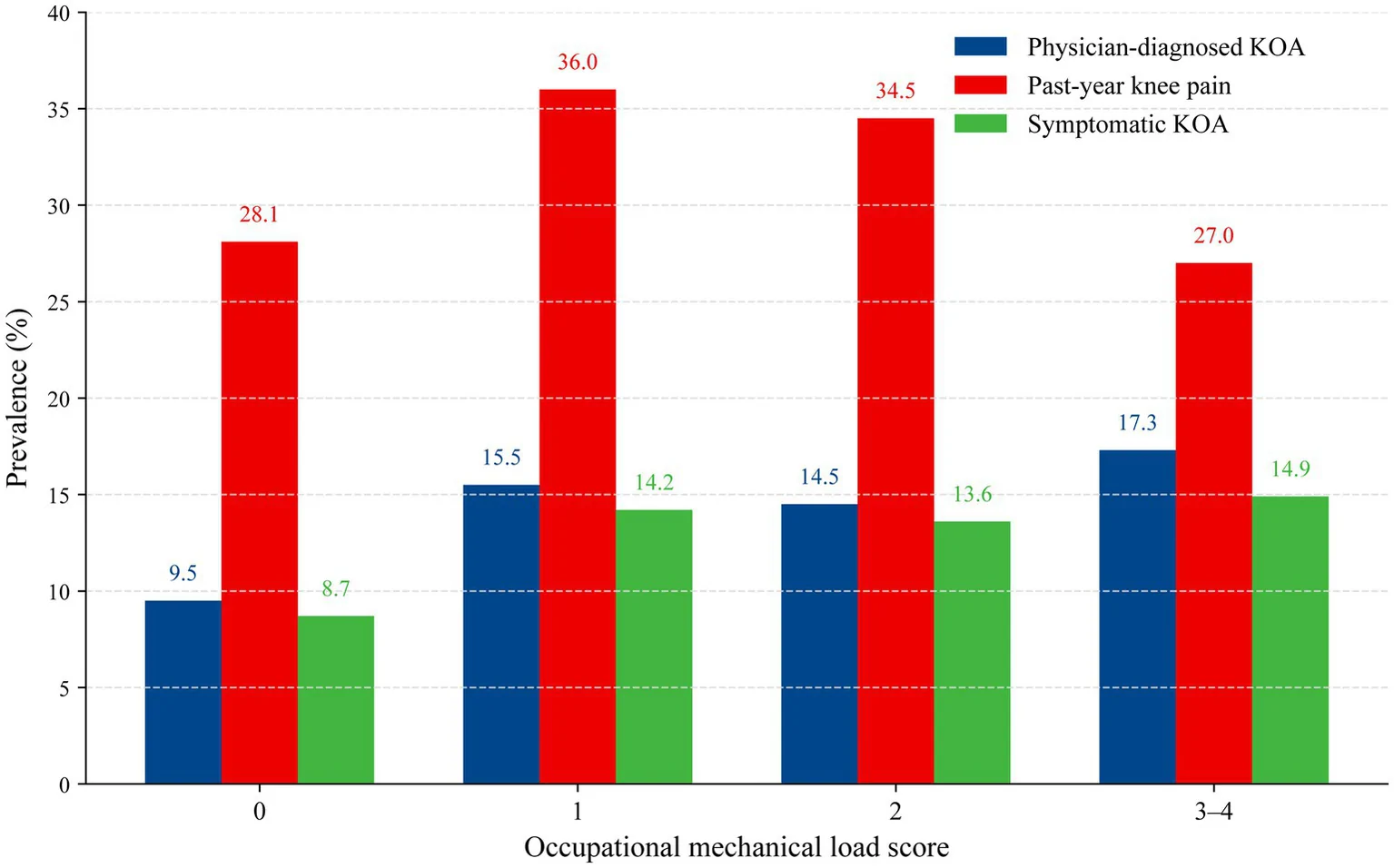
Burden of knee osteoarthritis and knee pain by occupational mechanical load score.

The prevalence of knee outcomes also differed by occupational mechanical load. Physician-diagnosed knee osteoarthritis was lowest among participants with an occupational mechanical load score of 0 (9.5, 95% CI 8.0–11.4) and highest among those with a score of 3–4 (17.3%, 14.0–21.1). Symptomatic knee osteoarthritis increased from 8.7% (7.2–10.4) in the score 0 group to 14.9% (11.8–18.6) in the score 3–4 group. Past-year knee pain was 28.1% (25.6–30.8) among participants with a score of 0, 36.0% (30.2–42.2) among those with a score of 1, 34.5% (26.3–43.8) among those with a score of 2, and 27.0% (22.9–31.4) among those with a score of 3–4 (Figure 4; Supplementary Table S2).

### 3.6 Factors associated with knee osteoarthritis and past-year knee pain

**Figure 5:**
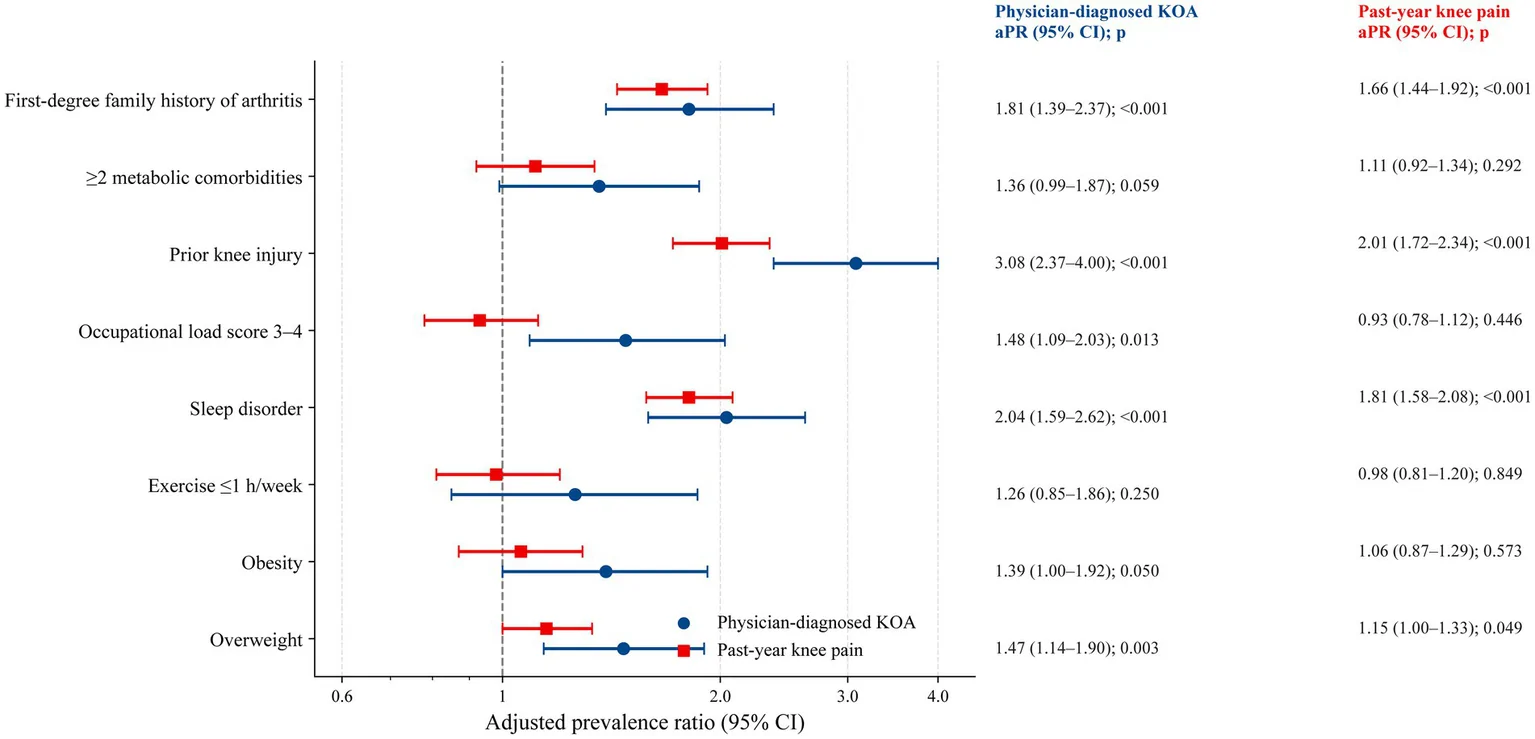
Visual summary of selected prespecified factors from the fully adjusted models for self-reported physician-diagnosed knee osteoarthritis and past-year knee pain. The complete model results are reported in Table 3; the figure is retained to facilitate comparison of effect magnitude and precision. Error bars show 95% CIs. aPR = adjusted prevalence ratio; KOA = knee osteoarthritis.

In the fully adjusted modified Poisson models, age showed consistent associations with all three outcomes (Table 3; Figure 5). Each 10-year increase in age was associated with physician-diagnosed KOA (aPR 1.27, 95% CI 1.12–1.44), past-year knee pain (1.19, 1.11–1.27), and symptomatic KOA (1.29, 1.12–1.48; all *p* < 0.001). Female sex was clearly associated with past-year knee pain (aPR 1.73, 95% CI 1.43–2.08; *p* < 0.001). Estimates for physician-diagnosed KOA (1.36, 1.00–1.86; *p* = 0.050) and symptomatic KOA (1.40, 1.01–1.94; *p* = 0.046) were less precise, with lower confidence limits close to 1. Variance inflation factors were all below 2.4, providing no indication of problematic statistical multicollinearity.

**Table 3:** Adjusted associations of selected demographic, clinical, lifestyle, and occupational factors with knee osteoarthritis and knee pain.

Predictor	Physician-diagnosed KOA	Past-year knee pain	Symptomatic KOA
	aPR (95% CI); *p* value	aPR (95% CI); *p* value	aPR (95% CI); *p* value
Age (per 10 years)	1.27 (1.12–1.44); *p* < 0.001	1.19 (1.11–1.27); *p* < 0.001	1.29 (1.12–1.48); *p* < 0.001
Female sex	1.36 (1.00–1.86); *p* = 0.050	1.73 (1.43–2.08); *p* < 0.001	1.40 (1.01–1.94); *p* = 0.046
Overweight	1.47 (1.14–1.90); *p* = 0.003	1.15 (1.00–1.33); *p* = 0.049	1.54 (1.17–2.02); *p* = 0.002
Obesity	1.39 (1.00–1.92); *p* = 0.050	1.06 (0.87–1.29); *p* = 0.573	1.43 (1.01–2.02); *p* = 0.044
Exercise 1–2.5 h/week	1.15 (0.78–1.71); *p* = 0.484	0.95 (0.77–1.16); *p* = 0.591	1.05 (0.70–1.57); *p* = 0.821
Exercise ≤1 h/week	1.26 (0.85–1.86); *p* = 0.250	0.98 (0.81–1.20); *p* = 0.849	1.17 (0.79–1.75); *p* = 0.435
Sleep disorder	2.04 (1.59–2.62); *p* < 0.001	1.81 (1.58–2.08); *p* < 0.001	2.32 (1.77–3.03); *p* < 0.001
Irregular routine	0.80 (0.61–1.05); *p* = 0.112	1.02 (0.89–1.17); *p* = 0.766	0.84 (0.63–1.12); *p* = 0.228
Occupational load score 1	1.29 (0.91–1.83); *p* = 0.153	0.99 (0.82–1.20); *p* = 0.929	1.27 (0.87–1.84); *p* = 0.215
Occupational load score 2	1.29 (0.77–2.15); *p* = 0.339	0.92 (0.70–1.20); *p* = 0.524	1.34 (0.79–2.26); *p* = 0.274
Occupational load score 3–4	1.48 (1.09–2.03); *p* = 0.013	0.93 (0.78–1.12); *p* = 0.446	1.39 (1.00–1.91); *p* = 0.048
Prior knee injury	3.08 (2.37–4.00); *p* < 0.001	2.01 (1.72–2.34); *p* < 0.001	2.97 (2.25–3.91); *p* < 0.001
One metabolic comorbidity	1.01 (0.78–1.31); *p* = 0.963	1.01 (0.87–1.17); *p* = 0.881	1.03 (0.79–1.36); *p* = 0.811
≥2 metabolic comorbidities	1.36 (0.99–1.87); *p* = 0.059	1.11 (0.92–1.34); *p* = 0.292	1.37 (0.98–1.92); *p* = 0.069
First-degree family history of arthritis	1.81 (1.39–2.37); *p* < 0.001	1.66 (1.44–1.92); *p* < 0.001	1.94 (1.47–2.57); *p* < 0.001

Adjusted prevalence ratios (aPRs) were estimated using modified Poisson regression with robust standard errors. The same fully adjusted model was used for each outcome and included age, sex, ethnicity (Han or other), residence, education, personal monthly income, BMI category, smoking, weekly exercise, sleep disorder, routine regularity, occupational mechanical load, previous knee injury, metabolic comorbidity count, and first-degree family history of arthritis. Reference categories are defined in the methods. *p* values are unadjusted; a Benjamini–Hochberg false-discovery-rate sensitivity analysis was undertaken across the 45 displayed comparisons. KOA = knee os.

Compared with normal BMI, overweight was associated with physician-diagnosed KOA (aPR 1.47, 95% CI 1.14–1.90; *p* = 0.003) and symptomatic KOA (1.54, 1.17–2.02; *p* = 0.002). The estimate for past-year knee pain was smaller and imprecise (1.15, 1.00–1.33; *p* = 0.049). For obesity, estimates for physician-diagnosed KOA (1.39, 1.00–1.92; *p* = 0.050) and symptomatic KOA (1.43, 1.01–2.02; *p* = 0.044) also had lower confidence limits close to 1, and there was no clear association with past-year knee pain (1.06, 0.87–1.29; *p* = 0.573; Table 3).

Sleep disorder showed comparatively large and precise associations with physician-diagnosed KOA (aPR 2.04, 95% CI 1.59–2.62), past-year knee pain (1.81, 1.58–2.08), and symptomatic KOA (2.32, 1.77–3.03; all *p* < 0.001). An occupational mechanical load score of 3–4 was associated with physician-diagnosed KOA (aPR 1.48, 95% CI 1.09–2.03; *p* = 0.013). The estimate for symptomatic KOA was weaker and borderline (1.39, 1.00–1.91; *p* = 0.048), and there was no association with past-year knee pain (0.93, 0.78–1.12; *p* = 0.446; Table 3; Figure 5).

Previous knee injury and first-degree family history of arthritis showed the most consistent associations across outcomes. Previous knee injury was associated with physician-diagnosed KOA (aPR 3.08, 95% CI 2.37–4.00), past-year knee pain (2.01, 1.72–2.34), and symptomatic KOA (2.97, 2.25–3.91; all *p* < 0.001). Corresponding estimates for first-degree family history were 1.81 (1.39–2.37), 1.66 (1.44–1.92), and 1.94 (1.47–2.57; all *p* < 0.001). Estimates for ≥2 metabolic comorbidities were compatible with both no association and modestly increased prevalence for physician-diagnosed KOA (1.36, 0.99–1.87; *p* = 0.059) and symptomatic KOA (1.37, 0.98–1.92; *p* = 0.069), and no clear association was observed for past-year knee pain (1.11, 0.92–1.34; *p* = 0.292; Table 3; Figure 5).

### 3.7 Factors associated with functional burden among participants with past-year knee pain

Among participants with past-year knee pain, several factors were associated with greater functional burden. Previous knee injury was associated with knee pain affecting sleep (aPR 1.57, 95% CI 1.27–1.93; *p* < 0.001), affecting work or daily activities (1.30, 1.11–1.52; *p* = 0.001), medical care seeking (1.39, 1.22–1.58; *p* < 0.001), and any sleep or work/activity impact (1.28, 1.10–1.48; *p* = 0.001). A high occupational mechanical load score of 3–4 was associated with sleep impact (aPR 1.42, 95% CI 1.08–1.85; *p* = 0.011), work or activity impact (1.44, 1.18–1.75; *p* < 0.001), medical care seeking (1.21, 1.02–1.43; *p* = 0.025), and any sleep or work/activity impact (1.41, 1.17–1.71; *p* < 0.001; Supplementary Table S3).

Higher BMI was also related to functional consequences of knee pain. Obesity was associated with work or activity impact (aPR 1.45, 95% CI 1.15–1.83; *p* = 0.002), medical care seeking (1.27, 1.07–1.52; *p* = 0.008), and any sleep or work/activity impact (1.37, 1.10–1.70; *p* = 0.005). Overweight showed similar associations with work or activity impact (aPR 1.40, 95% CI 1.19–1.66; *p* < 0.001), medical care seeking (1.17, 1.01–1.36; *p* = 0.042), and any sleep or work/activity impact (1.37, 1.17–1.61; *p* < 0.001). Sleep disorder was associated with sleep impact (aPR 1.43, 95% CI 1.17–1.76; *p* < 0.001), work or activity impact (1.22, 1.05–1.42; *p* = 0.011), and any sleep or work/activity impact (1.33, 1.15–1.54; *p* < 0.001). Two or more metabolic comorbidities were associated with all functional-impact outcomes, including sleep impact (aPR 1.46, 95% CI 1.12–1.89; *p* = 0.004), work or activity impact (1.44, 1.17–1.78; *p* < 0.001), medical care seeking (1.24, 1.04–1.48; *p* = 0.017), and any sleep or work/activity impact (1.35, 1.11–1.65; *p* = 0.003; Supplementary Table S3).

### 3.8 Sensitivity analyses

The principal patterns were similar across data-quality and population-restriction analyses. After exclusion of responses completed in less than 180 s, estimates for physician-diagnosed KOA remained similar for sleep disorder (aPR 1.97, 95% CI 1.54–2.53), occupational load score 3–4 (1.52, 1.11–2.09), and previous knee injury (3.02, 2.33–3.92). Results were also broadly unchanged after exclusion of responses completed in less than 300 s, restriction to a likely Urumqi source, exclusion of logical conflicts, and analysis of all adults aged ≥18 years (Supplementary Table S4). In the analysis restricted to participants aged ≥45 years (*n* = 1,636; 206 physician-diagnosed KOA, 480 past-year knee-pain, and 185 symptomatic-KOA events), estimates remained similar for previous knee injury (aPRs 3.12, 1.89, and 2.97, respectively), sleep disorder (2.14, 1.74, and 2.41), and first-degree family history of arthritis (1.74, 1.62, and 1.93). The false-discovery-rate sensitivity analysis supported the principal associations with age, sleep disorder, previous knee injury, family history, and overweight for physician-diagnosed and symptomatic KOA; several estimates with unadjusted *p* values near 0.05 did not remain below the 5% false-discovery-rate threshold. Exploratory log-binomial models did not converge reliably across all outcomes.

## 4 Discussion

In this community-based survey of adults aged 40 years or older in Urumqi, knee osteoarthritis and knee pain were common and were accompanied by substantial functional burden. Approximately one in eight participants reported physician-diagnosed knee osteoarthritis, nearly three in ten reported knee pain in the past year, and one in nine met our definition of symptomatic knee osteoarthritis. Among participants with past-year knee pain, more than 40% reported sleep disturbance, more than half reported interference with work or daily activities, and more than 60% had sought medical care. These findings indicate that knee osteoarthritis and knee pain in this setting are not only musculoskeletal diagnoses, but also common chronic health problems that affect sleep, mobility, work capacity, and use of primary-care services.

The prevalence observed in our study is broadly consistent with previous evidence from China and international studies, although comparisons should be made with caution because of differences in definitions, age structure, and sampling methods. In the China Health and Retirement Longitudinal Study, symptomatic knee osteoarthritis affected 8.1% of adults aged 45 years or older, with higher prevalence in women, older adults, less educated groups, and some western and southwestern regions (15). A meta-analysis of Chinese studies also showed a substantial burden of symptomatic knee osteoarthritis, particularly among women and older individuals (16). Our estimate of symptomatic knee osteoarthritis was somewhat higher than that reported in some national datasets, but it remains plausible for a middle-aged and older community sample in northwest China. At the global level, the GBD 2021 analysis estimated that 595 million people were living with osteoarthritis in 2020, with knee osteoarthritis the most common anatomical site and a marked increase projected by 2050 (17). Analyses covering 204 countries and territories similarly showed that the prevalence, incidence, and years lived with disability attributable to knee osteoarthritis have increased over recent decades, with high BMI contributing an increasing share of disease burden (4). Our findings therefore reflect a local manifestation of a broader transition: musculoskeletal disability is becoming an increasingly important component of chronic disease burden in ageing and urbanising populations.

A key feature of this study is the distinction between self-reported physician-diagnosed knee osteoarthritis, past-year knee pain, and symptomatic knee osteoarthritis. The symptom burden was greater than the burden captured by physician-diagnosis history alone. This distinction is relevant to clinical and public health interpretation, but it also highlights the limitations of survey-based definitions. Participants with knee pain who had not sought care or received a diagnosis were classified as not having physician-diagnosed knee osteoarthritis, whereas past-year knee pain could reflect conditions other than knee osteoarthritis. Diagnostic history may also vary with access to care, health awareness, and recall. The differences between these outcomes should therefore not be interpreted as evidence of an undiagnosed-disease gap without clinical confirmation. Previous studies indicate that pain and high-impact symptoms are closely related to physical function, disability, and health-care use (5), while the accuracy of self-reported arthritis is acceptable but imperfect in large epidemiological studies (8). Our findings support reporting diagnostic history and symptom-related impact as complementary, rather than interchangeable, measures.

The high proportions of participants reporting exercise-related and cold-weather knee pain should be interpreted descriptively. Exercise-related pain is not evidence that physical activity is harmful; it may instead identify people who need individualised guidance on safe, progressive activity. Exercise is recommended in major osteoarthritis guidelines and improves pain and function in intervention studies (18–20). In Urumqi, long cold seasons might influence symptom perception and outdoor activity, but the cross-sectional questionnaire cannot establish that cold exposure caused knee pain or activity avoidance. These observations could inform the design of future prospective studies and locally adapted symptom-management programmes, including indoor exercise options and seasonal advice, but they do not demonstrate the effectiveness of such strategies.

The associations between age, sex, and knee outcomes were consistent with established epidemiological evidence. Older age was associated with physician-diagnosed knee osteoarthritis, past-year knee pain, and symptomatic knee osteoarthritis. Women had a substantially higher prevalence of past-year knee pain and symptomatic knee osteoarthritis than men. Similar patterns have been reported in global and Chinese studies, in which women and older adults carry a disproportionate burden of knee osteoarthritis (4, 15–17). The sex difference is likely to reflect a combination of biological, biomechanical, and social factors, including differences in body composition, muscle strength, pain perception, health-care seeking, lifetime physical workload, and post-menopausal changes. Because our study did not measure hormonal status, joint alignment, or muscle strength, these mechanisms could not be examined directly. Nevertheless, the findings support the need for sex-sensitive prevention and management strategies, especially for middle-aged and older women.

Higher BMI was associated with physician-diagnosed and symptomatic knee osteoarthritis in this study. Prospective meta-analyses and cohort studies have reported dose–response and longitudinal associations between adiposity and knee osteoarthritis (21, 22), and mechanical loading and metabolic inflammation are plausible mechanisms described in previous work (23). However, our cross-sectional data cannot determine whether excess body weight preceded knee symptoms, whether pain-related activity restriction contributed to weight gain, or whether both reflected shared determinants. Metabolic comorbidity showed weaker and less precise associations than BMI. Because BMI and metabolic conditions are interrelated, the mutually adjusted coefficients should be interpreted as conditional associations rather than independent causal effects. Although variance inflation factors did not indicate problematic collinearity, residual confounding, mediation, and common-cause pathways cannot be separated in this design.

Previous knee injury had the largest adjusted prevalence ratios for all three knee outcomes. This pattern is consistent with longitudinal and meta-analytic evidence linking traumatic knee injury with later osteoarthritis (24, 25). Nevertheless, the survey did not capture the timing, anatomical diagnosis, severity, or treatment of injury, and participants with current knee problems might recall previous injuries more readily. The observed associations therefore support previous knee injury as a clinically relevant marker, but do not quantify the causal effect of injury or the benefit of any specific prevention or rehabilitation strategy.

A higher study-specific occupational mechanical load score was associated with physician-diagnosed and symptomatic knee osteoarthritis, and, among participants with knee pain, with several functional-impact outcomes. Previous studies have linked kneeling, squatting, climbing, lifting, and prolonged standing with knee osteoarthritis (26). However, our score measured current rather than lifetime work requirements and was not a validated cumulative exposure instrument. Its lack of an independent association with past-year knee pain, together with the non-monotonic descriptive pattern across score categories, argues against a simple dose–response interpretation. Reverse selection is also possible because people with more severe symptoms may reduce demanding tasks or leave physically intensive work. The results should therefore be interpreted as cross-sectional occupational correlates, not as evidence that the measured work activities caused the outcomes.

Sleep disorder was associated with all three knee outcomes and with several functional-impact measures. This relationship is likely to be complex and potentially bidirectional: pain can disrupt sleep, whereas poor sleep can heighten pain sensitivity, fatigue, and distress. Systematic reviews and longitudinal studies support links between sleep and osteoarthritis symptoms (27–29), but the single self-reported sleep item and cross-sectional timing in our study do not permit temporal ordering. Sleep disorder should therefore be viewed as a marker of greater concurrent symptom burden and a candidate for further longitudinal assessment, rather than as a demonstrated cause of knee osteoarthritis or knee pain.

The findings have potential implications for community assessment, but they should not be read as evidence that modifying the associated factors identified here will necessarily reduce knee osteoarthritis or knee pain. Asking about past-year knee pain, pain-related sleep or activity impact, previous knee injury, body size, sleep problems, and occupational demands could help characterise symptom burden and identify groups for further clinical assessment. Education, exercise, and weight management are recommended as core non-surgical treatments on the basis of external guideline and intervention evidence (18, 19), not on the basis of causal effects estimated in this survey. The high proportion of participants who had sought medical care suggests a need to examine whether existing primary-care pathways provide timely assessment, appropriate exercise advice, and referral for persistent or disabling symptoms.

This study has several strengths. It used a large community-recruited sample from Urumqi and captured both diagnostic history and symptom-related burden, providing a broader description than prevalence of physician diagnosis alone. The questionnaire included anthropometric, sleep, injury, comorbidity, family-history, and occupational characteristics, and the analyses distinguished physician-diagnosed knee osteoarthritis, past-year knee pain, symptomatic knee osteoarthritis, and functional outcomes among participants with pain. Duplicate removal, prespecified data-quality restrictions, sensitivity analyses, and explicit collinearity assessment supported the consistency and transparency of the findings.

Several limitations should be acknowledged. First, the cross-sectional design precludes causal or temporal inference. In particular, the direction of the associations involving sleep, BMI, metabolic comorbidity, physical activity, and occupational load cannot be established, and these variables may act as confounders, intermediates, or consequences of pain. Although variance inflation factors were low, statistical collinearity diagnostics do not resolve these causal relationships, and residual confounding by depression, pain catastrophising, medication use, physical capacity, and other unmeasured characteristics remains possible. Second, KOA was based on self-reported physician-diagnosis history and was not confirmed by radiography, clinical examination, or medical records. This definition might underestimate prevalence among symptomatic participants who had not sought care or received a diagnosis, but misunderstanding or inaccurate recall could also lead to over-reporting. Past-year knee pain may be more sensitive but less specific because it can arise from conditions other than osteoarthritis. If misclassification was unrelated to the associated factors, prevalence-ratio estimates would generally be expected to move towards the null; however, differential diagnosis or reporting—for example, greater clinical contact among participants with previous injury, obesity, or occupational symptoms—could bias estimates in either direction. Third, height, weight, injury, comorbidities, work demands, sleep, and functional impact were self-reported and are subject to recall and reporting error. The questionnaire did not collect analgesic use, topical treatment, injections, or other knee-pain therapies, which limited interpretation of symptom severity and health-care seeking. Fourth, the primary and secondary analyses involved multiple comparisons. Although the main exposure–outcome comparisons were prespecified and a false-discovery-rate sensitivity analysis was added, findings with confidence intervals close to 1 should remain hypothesis-generating. The primary models converged with 212–562 events, but the symptomatic-KOA model and conditional functional-impact analyses had lower information per parameter; their estimates should therefore be interpreted with attention to precision rather than *p* values alone. Fifth, online and community recruitment may have over-represented urban, educated, digitally connected, or health-conscious residents; the results are survey-based estimates and should not be interpreted as population-representative prevalence for all adults in Urumqi. Finally, the survey did not measure pain intensity, radiographic severity, mood symptoms, knee alignment, muscle strength, inflammatory markers, objective physical activity, or lifetime occupational exposure, and ethnicity was grouped as Han or other, limiting more detailed assessment of population heterogeneity.

## 5 Conclusion

Knee osteoarthritis and knee pain were common among community-recruited adults aged 40 years or older in Urumqi and were frequently accompanied by sleep disturbance, activity limitation, and health-care seeking. Previous knee injury, sleep disorder, higher BMI, family history of arthritis, and occupational mechanical load were associated with one or more knee outcomes, but these cross-sectional estimates do not establish temporal sequence or causality. The findings provide locally relevant survey evidence for community assessment and hypothesis generation; longitudinal and intervention studies are needed to determine whether addressing these factors can reduce symptoms or disability.

## Data Availability

The raw data supporting the conclusions of this article will be made available by the authors, without undue reservation.
