# Supplementary material for: Prevalence, functional impact, and associated factors of knee osteoarthritis and knee pain among adults in Urumqi, Northwest China: a community-based cross-sectional study
Source: Front Public Health. 2026 Sep 11;14:1903768. doi: 10.3389/fpubh.2026.1903768 (PMC13612206; doi:10.3389/fpubh.2026.1903768)
Supplement: Supplementary file 1 [file Data_Sheet_1.docx]

**SUPPLEMENTARY FILE S1**

English Translation of the Original Questionnaire

Survey of the Prevalence and Risk Factors of Knee Osteoarthritis among Residents of Urumqi

*Questionnaire on the Risk, Treatment, Prognosis, and Health Management of Knee Osteoarthritis*

*Translator’s note. This document is a faithful English translation of the blank Chinese questionnaire used in the study. Item numbering, response options, and skip instructions have been retained. The English version is supplied for transparency and was not used to collect data or independently validated for administration in English. Minor typographical artefacts in the exported Chinese form were normalised without changing the intended meaning. The original form labels two sections as “Part III”; this numbering is retained below. One liang is equivalent to 50 g. An asterisk indicates a required item.*

**Blank questionnaire: no participant responses are included.**

# Participant Information

Dear participant,

You are invited to take part in a survey entitled “Questionnaire on the Risk, Treatment, Prognosis, and Health Management of Knee Osteoarthritis”. The study is intended to improve understanding of factors associated with the onset and prognosis of knee osteoarthritis, explore suitable health-management pathways, and support better recovery and disease management.

The questionnaire contains six parts, including general demographic information, lifestyle and dietary habits, and screening of factors related to knee osteoarthritis. The information collected will be used to describe participants’ health-management status and to inform the development of more effective health-management measures.

The questionnaire takes approximately 30 minutes to complete. Please answer each question according to your actual circumstances. All information will be kept strictly confidential and used only for research. Participation is voluntary; you may withdraw at any time, and doing so will not adversely affect your usual medical care.

# Part I. General Demographic Information

**1. Name** *[Open-ended] **

________________________________________________________________

**2. Telephone number** *[Open-ended] **

________________________________________________________________

**3. Date of birth: ____ year ____ month ____ day** *[Open-ended] **

**4. Sex** *[Single choice] **

| ( ) Male | ( ) Female |
| --- | --- |

**5. Ethnicity** *[Single choice] **

| ( ) Han | ( ) Uyghur |
| --- | --- |
| ( ) Kazakh | ( ) Manchu |
| ( ) Korean | ( ) Hui |
| ( ) Other: __________ |  |

**6. Usual place of residence** *[Single choice] **

| ( ) Urban | ( ) Rural | ( ) Suburban |
| --- | --- | --- |

**7. Highest level of education** *[Single choice] **

| ( ) No formal schooling/illiterate | ( ) Primary school |
| --- | --- |
| ( ) Junior high school | ( ) Senior high school |
| ( ) College/university | ( ) Master’s degree or above |

**8. Occupation** *[Single choice] **

| ( ) Civil servant | ( ) Professional/technical worker |
| --- | --- |
| ( ) Office clerk | ( ) Teacher |
| ( ) Corporate manager | ( ) Manual worker |
| ( ) Farmer | ( ) Student |
| ( ) Active-duty military personnel | ( ) Freelancer |
| ( ) Self-employed business owner | ( ) Unemployed |
| ( ) Other: __________ |  |

**9. Personal monthly income** *[Single choice] **

| ( ) CNY 1000–3000 | ( ) CNY 3000–5000 |
| --- | --- |
| ( ) CNY 5000–8000 | ( ) CNY 8000–10 000 |
| ( ) More than CNY 10 000 |  |

**10. Marital status** *[Single choice] **

| ( ) Never married | ( ) Married |
| --- | --- |
| ( ) Widowed | ( ) Divorced |
| ( ) Remarried |  |

**11. Living arrangement** *[Multiple choice] **

| [ ] Living alone | [ ] Living with parents |
| --- | --- |
| [ ] Living with spouse/partner | [ ] Living with children |
| [ ] Living with a caregiver/domestic helper | [ ] Other: __________ |

**12. Home address** *[Open-ended] **

________________________________________________________________

**13. Main method(s) of paying for medical care** *[Multiple choice] **

| [ ] Urban Employee Basic Medical Insurance | [ ] Basic Medical Insurance for Urban Residents |
| --- | --- |
| [ ] New Rural Cooperative Medical Scheme | [ ] Medical assistance for low-income households |
| [ ] Commercial health insurance | [ ] Fully publicly funded care |
| [ ] Fully self-funded care | [ ] Other social insurance |
| [ ] Other: __________ |  |

**14. Who provided the information for this survey?** *[Single choice] **

| ( ) The participant | ( ) Legal guardian | ( ) Family member |
| --- | --- | --- |

**15. Height (cm)** *[Open-ended] **

______________________________

**16. Weight (kg)** *[Open-ended] **

______________________________

# Part II. Knee Osteoarthritis and Knee Pain

**17. Were you first diagnosed with knee osteoarthritis during the past 12 months?** *[Single choice] **

| ( ) Yes | ( ) No |
| --- | --- |

**18. Have you ever been explicitly diagnosed with knee osteoarthritis by a physician?** *[Single choice] **

| ( ) Yes | ( ) No |
| --- | --- |

**19. Have you had knee pain during the past 12 months?** *[Single choice] **

| ( ) Yes | ( ) No |
| --- | --- |

**20. Have you ever had knee pain previously?** *[Single choice] * If “No”, skip to Question 28.*

| ( ) Yes | ( ) No |
| --- | --- |

**21. Has your knee pain affected your sleep?** *[Single choice] **

| ( ) Yes | ( ) No |
| --- | --- |

**22. Has your knee pain affected your work or other activities?** *[Single choice] **

| ( ) Yes | ( ) No |
| --- | --- |

**23. Have you sought medical help because of knee pain?** *[Single choice] **

| ( ) Yes | ( ) No |
| --- | --- |

**24. Have you ever experienced knee pain after exercise?** *[Single choice] * If “No”, skip to Question 26.*

| ( ) Yes | ( ) No |
| --- | --- |

**25. During the past 12 months, have you experienced knee pain after exercise?** *[Single choice] **

| ( ) Yes | ( ) No |
| --- | --- |

**26. Have you ever experienced knee pain in cold weather?** *[Single choice] * If “No”, skip to Question 28.*

| ( ) Yes | ( ) No |
| --- | --- |

**27. During the past 12 months, have you experienced knee pain in cold weather?** *[Single choice] **

| ( ) Yes | ( ) No |
| --- | --- |

**28. Have you ever sustained a knee injury?** *[Single choice] * If “No”, skip to Question 30.*

| ( ) Yes | ( ) No |
| --- | --- |

**29. During the past 12 months, have you sustained a knee injury?** *[Single choice] **

| ( ) Yes | ( ) No |
| --- | --- |

# Part III. Lifestyle and Occupational Characteristics

**30. Which statement best describes your smoking during the past year?** *[Single choice] **

| ( ) Never smoked | ( ) Stopped smoking more than 1 year ago |
| --- | --- |
| ( ) Stopped smoking less than 1 year ago | ( ) Currently smoke 1–10 cigarettes per day |
| ( ) Currently smoke 10–20 cigarettes per day | ( ) Currently smoke more than 20 cigarettes per day |

**31. How would you describe your salt intake during the past month? (One full beer-bottle cap is approximately 6 g of salt.)** *[Single choice] **

( ) High-salt taste; >11 g/day; regularly eat pickled or preserved foods and do not restrict salt

( ) High-salt taste; >11 g/day; do not eat pickled/preserved foods, soda crackers, or other high-salt foods

( ) Actively try to reduce salt, but sometimes order food because of convenience

( ) Actively control salt intake to approximately 8–10 g/day

( ) Actively control salt intake to approximately 3–7 g/day

**32. During the past month, approximately how much water did you drink each day? (One bottle of mineral water = 550 mL.)** *[Single choice] **

| ( ) <100 mL | ( ) 100–500 mL |
| --- | --- |
| ( ) 500–1000 mL | ( ) 1000–1500 mL |
| ( ) >1500 mL |  |

**33. How much time do you spend on physical exercise each week? Regular exercise was defined in the original questionnaire as at least 2.5 hours per week of regular moderate-intensity activity that makes you feel warm or slightly out of breath.** *[Single choice] **

| ( ) 1 hour or less per week | ( ) 1–2.5 hours per week | ( ) 2.5 hours or more per week |
| --- | --- | --- |

**34. Do you have a sleep disorder?** *[Single choice] **

| ( ) Yes | ( ) No |
| --- | --- |

**35. Is your daily routine regular?** *[Single choice] **

| ( ) Yes | ( ) No |
| --- | --- |

**36. What is the main nature of your work?** *[Single choice] **

| ( ) Office-based work | ( ) Manual labour |
| --- | --- |
| ( ) Regularly working outdoors | ( ) Other: __________ |

**37. Does your work require prolonged standing or walking?** *[Single choice] * If “No”, skip to Question 39.*

| ( ) Yes | ( ) No |
| --- | --- |

**38. If yes, approximately how long do you stand or walk each day?** *[Single choice] **

| ( ) Less than 2 hours | ( ) 2–4 hours |
| --- | --- |
| ( ) 4–6 hours | ( ) More than 6 hours |

**39. Does your work require frequent bending or twisting of the knee?** *[Single choice] **

| ( ) Yes | ( ) No |
| --- | --- |

**40. Does your work require repeated squatting or stair climbing?** *[Single choice] **

| ( ) Yes | ( ) No |
| --- | --- |

**41. Does your work require handling or carrying heavy loads?** *[Single choice] **

| ( ) Yes | ( ) No |
| --- | --- |

## Patient Assessment of Constipation Symptoms (PAC-SYM)

Please recall your physical condition during the past 2 weeks and rate the severity of each symptom: 0=none/normal, 1=mild, 2=moderate, 3=severe, and 4=very severe.

**42. Rate the severity of each constipation-related symptom during the past 2 weeks.** *[Matrix single choice] **

| **Symptom** | **None/normal (0)** | **Mild (1)** | **Moderate (2)** | **Severe (3)** | **Very severe (4)** |
| --- | --- | --- | --- | --- | --- |
| Hard stools | ○ | ○ | ○ | ○ | ○ |
| Small stool volume | ○ | ○ | ○ | ○ | ○ |
| Reduced bowel-movement frequency | ○ | ○ | ○ | ○ | ○ |
| Straining during bowel movements | ○ | ○ | ○ | ○ | ○ |
| Painful bowel movements | ○ | ○ | ○ | ○ | ○ |
| Feeling of incomplete evacuation | ○ | ○ | ○ | ○ | ○ |
| Urge to defecate but difficulty passing stool | ○ | ○ | ○ | ○ | ○ |
| Rectal/anal bleeding or tearing | ○ | ○ | ○ | ○ | ○ |
| Rectal/anal burning | ○ | ○ | ○ | ○ | ○ |
| Stomach pain | ○ | ○ | ○ | ○ | ○ |
| Abdominal cramping | ○ | ○ | ○ | ○ | ○ |
| Abdominal bloating/distension | ○ | ○ | ○ | ○ | ○ |

## Patient Assessment of Constipation Quality of Life (PAC-QOL)

**43. During the past 2 weeks, how severe or intense were the following constipation-related symptoms?** *[Matrix single choice] **

| **Item** | **Not at all** | **A little** | **Moderate** | **Quite severe** | **Extremely severe** |
| --- | --- | --- | --- | --- | --- |
| Felt bloated | ○ | ○ | ○ | ○ | ○ |
| Felt heavy | ○ | ○ | ○ | ○ | ○ |

**44. During the past 2 weeks, how often did constipation make you feel the following?** *[Matrix single choice] **

| **Item** | **Never** | **Rarely** | **Sometimes** | **Most of the time** | **Always** |
| --- | --- | --- | --- | --- | --- |
| Felt physically uncomfortable | ○ | ○ | ○ | ○ | ○ |
| Had the urge to defecate but had difficulty passing stool | ○ | ○ | ○ | ○ | ○ |
| Felt uncomfortable around other people | ○ | ○ | ○ | ○ | ○ |
| Ate less because of constipation | ○ | ○ | ○ | ○ | ○ |

**45. During the past 2 weeks, how severe or intense were the following effects of constipation on daily life?** *[Matrix single choice] **

| **Item** | **Not at all** | **A little** | **Moderate** | **Quite severe** | **Extremely severe** |
| --- | --- | --- | --- | --- | --- |
| Had to carefully consider what to eat | ○ | ○ | ○ | ○ | ○ |
| Loss of appetite | ○ | ○ | ○ | ○ | ○ |
| Worried about not being able to choose food freely (e.g., at a friend’s home) | ○ | ○ | ○ | ○ | ○ |
| Felt uncomfortable when away from home because of spending too long in the bathroom | ○ | ○ | ○ | ○ | ○ |
| Felt uncomfortable when away from home because of frequent bathroom visits | ○ | ○ | ○ | ○ | ○ |
| Constantly worried about changing lifestyle habits (e.g., travel or going out) | ○ | ○ | ○ | ○ | ○ |

**46. During the past 2 weeks, how often did you experience the following feelings related to constipation?** *[Matrix single choice] **

| **Item** | **Never** | **Rarely** | **Sometimes** | **Most of the time** | **Always** |
| --- | --- | --- | --- | --- | --- |
| Felt irritable | ○ | ○ | ○ | ○ | ○ |
| Felt uneasy or restless | ○ | ○ | ○ | ○ | ○ |
| Felt constantly bothered | ○ | ○ | ○ | ○ | ○ |
| Felt tense | ○ | ○ | ○ | ○ | ○ |
| Felt lacking in confidence | ○ | ○ | ○ | ○ | ○ |
| Felt that life was out of control | ○ | ○ | ○ | ○ | ○ |

**47. During the past 2 weeks, how severe or intense were the following concerns related to constipation?** *[Matrix single choice] **

| **Item** | **Not at all** | **A little** | **Moderate** | **Quite severe** | **Extremely severe** |
| --- | --- | --- | --- | --- | --- |
| Worried because you did not know when you would have a bowel movement | ○ | ○ | ○ | ○ | ○ |
| Worried that you would be unable to have a bowel movement | ○ | ○ | ○ | ○ | ○ |
| Constipation interfered with your life | ○ | ○ | ○ | ○ | ○ |

**48. During the past 2 weeks, how often did constipation affect daily life in the following ways?** *[Matrix single choice] **

| **Item** | **Never** | **Rarely** | **Sometimes** | **Most of the time** | **Always** |
| --- | --- | --- | --- | --- | --- |
| Worried that the condition would become worse | ○ | ○ | ○ | ○ | ○ |
| Felt that your body was unable to work | ○ | ○ | ○ | ○ | ○ |
| Had bowel movements less often than expected | ○ | ○ | ○ | ○ | ○ |

**49. During the past 2 weeks, how satisfied were you with the following?** *[Matrix single choice] **

| **Item** | **Very satisfied** | **Quite satisfied** | **Neutral** | **Somewhat dissatisfied** | **Very dissatisfied** |
| --- | --- | --- | --- | --- | --- |
| Frequency of bowel movements | ○ | ○ | ○ | ○ | ○ |
| Regularity of bowel movements | ○ | ○ | ○ | ○ | ○ |
| Your bowel function | ○ | ○ | ○ | ○ | ○ |
| Previous treatment | ○ | ○ | ○ | ○ | ○ |

# Simplified Food-Frequency Questionnaire

Please recall your dietary intake during the past month and answer each item as accurately as possible. Amounts are expressed in liang; 1 liang=50 g. The frequency and usual amount can be used to estimate the average daily intake of each food group.

**50. A1. How often did you consume cooked rice during the past month?** *[Single choice] **

| ( ) 0. Never | ( ) 1. Less than once per month | ( ) 2. 1–3 times per month |
| --- | --- | --- |
| ( ) 3. 1–2 times per week | ( ) 4. 3–4 times per week | ( ) 5. 5–6 times per week |
| ( ) 6. Once per day | ( ) 7. Twice per day | ( ) 8. Three or more times per day |

**51. A2. How much cooked rice did you usually consume each time?** *[Single choice] **

Portion-size reference: 2 liang of cooked rice (100 g).

| ( ) 1. 1 liang or less (≤50 g) | ( ) 2. 2 liang (100 g) | ( ) 3. 3 liang (150 g) |
| --- | --- | --- |
| ( ) 4. 4 liang (200 g) | ( ) 5. 5 liang or more (≥250 g) | ( ) 6. Did not consume |

**52. A3. How often did you consume congee, rice porridge, or rice soaked in water during the past month?** *[Single choice] **

| ( ) 0. Never | ( ) 1. Less than once per month | ( ) 2. 1–3 times per month |
| --- | --- | --- |
| ( ) 3. 1–2 times per week | ( ) 4. 3–4 times per week | ( ) 5. 5–6 times per week |
| ( ) 6. Once per day | ( ) 7. Twice per day | ( ) 8. Three or more times per day |

**53. A4. How much congee, rice porridge, or rice soaked in water did you usually consume each time?** *[Single choice] **

| ( ) 1. 1 liang or less (≤50 g) | ( ) 2. 2 liang (100 g) | ( ) 3. 3 liang (150 g) |
| --- | --- | --- |
| ( ) 4. 4 liang (200 g) | ( ) 5. 5 liang or more (≥250 g) | ( ) 6. Did not consume |

**54. A5. How often did you consume wheat-flour foods (steamed buns, bread, noodles, or flatbread) during the past month?** *[Single choice] **

| ( ) 0. Never | ( ) 1. Less than once per month | ( ) 2. 1–3 times per month |
| --- | --- | --- |
| ( ) 3. 1–2 times per week | ( ) 4. 3–4 times per week | ( ) 5. 5–6 times per week |
| ( ) 6. Once per day | ( ) 7. Twice per day | ( ) 8. Three or more times per day |

**55. A6. How much wheat-flour foods did you usually consume each time?** *[Single choice] **

Portion-size reference: 2 liang of steamed bun (100 g).

| ( ) 1. 1 liang or less (≤50 g) | ( ) 2. 2 liang (100 g) | ( ) 3. 3 liang (150 g) |
| --- | --- | --- |
| ( ) 4. 4 liang (200 g) | ( ) 5. 5 liang or more (≥250 g) | ( ) 6. Did not consume |

**56. A7. How often did you consume sweets, pastries, or cakes during the past month?** *[Single choice] **

| ( ) 0. Never | ( ) 1. Less than once per month | ( ) 2. 1–3 times per month |
| --- | --- | --- |
| ( ) 3. 1–2 times per week | ( ) 4. 3–4 times per week | ( ) 5. 5–6 times per week |
| ( ) 6. Once per day | ( ) 7. Twice per day | ( ) 8. Three or more times per day |

**57. A8. How much sweets, pastries, or cakes did you usually consume each time?** *[Single choice] **

| ( ) 1. 1 liang or less (≤50 g) | ( ) 2. 2 liang (100 g) | ( ) 3. 3 liang (150 g) |
| --- | --- | --- |
| ( ) 4. 4 liang (200 g) | ( ) 5. 5 liang or more (≥250 g) | ( ) 6. Did not consume |

**58. A9. How often did you consume fried foods (e.g., fried dough sticks or fried cakes) during the past month?** *[Single choice] **

| ( ) 0. Never | ( ) 1. Less than once per month | ( ) 2. 1–3 times per month |
| --- | --- | --- |
| ( ) 3. 1–2 times per week | ( ) 4. 3–4 times per week | ( ) 5. 5–6 times per week |
| ( ) 6. Once per day | ( ) 7. Twice per day | ( ) 8. Three or more times per day |

**59. A10. How much fried foods (e.g., fried dough sticks, fried cakes, French fries, fried chicken wings, fried chicken legs, or fried pork cutlets) did you usually consume each time?** *[Single choice] **

| ( ) 1. Half an item or less | ( ) 2. 1 item (approximately 2 liang/100 g) | ( ) 3. 3 liang (150 g) |
| --- | --- | --- |
| ( ) 4. 2 items (approximately 4 liang/200 g) | ( ) 5. 5 liang or more (≥250 g) | ( ) 6. Did not consume |

**60. A11. How often did you consume filled foods (e.g., baozi, wontons, or dumplings) during the past month?** *[Single choice] **

| ( ) 0. Never | ( ) 1. Less than once per month | ( ) 2. 1–3 times per month |
| --- | --- | --- |
| ( ) 3. 1–2 times per week | ( ) 4. 3–4 times per week | ( ) 5. 5–6 times per week |
| ( ) 6. Once per day | ( ) 7. Twice per day | ( ) 8. Three or more times per day |

**61. A12. How much filled foods did you usually consume each time?** *[Single choice] **

| ( ) 1. 1 liang or less (≤50 g) | ( ) 2. 2 liang (100 g) | ( ) 3. 3 liang (150 g) |
| --- | --- | --- |
| ( ) 4. 4 liang (200 g) | ( ) 5. 5 liang or more (≥250 g) | ( ) 6. Did not consume |

**62. A13. How often did you consume coarse or mixed grains (including brown rice, millet, corn, coix seed, oats, adzuki beans, and mung beans) during the past month?** *[Single choice] **

| ( ) 0. Never | ( ) 1. Less than once per month | ( ) 2. 1–3 times per month |
| --- | --- | --- |
| ( ) 3. 1–2 times per week | ( ) 4. 3–4 times per week | ( ) 5. 5–6 times per week |
| ( ) 6. Once per day | ( ) 7. Twice per day | ( ) 8. Three or more times per day |

**63. A14. How much coarse or mixed grains did you usually consume each time?** *[Single choice] **

| ( ) 1. 1 liang or less (≤50 g) | ( ) 2. 2 liang (100 g) | ( ) 3. 3 liang (150 g) |
| --- | --- | --- |
| ( ) 4. 4 liang (200 g) | ( ) 5. 5 liang or more (≥250 g) | ( ) 6. Did not consume |

**64. A15. How often did you consume tubers and root foods (including sweet potatoes, potatoes, taro, Chinese yam, and konjac) during the past month?** *[Single choice] **

| ( ) 0. Never | ( ) 1. Less than once per month | ( ) 2. 1–3 times per month |
| --- | --- | --- |
| ( ) 3. 1–2 times per week | ( ) 4. 3–4 times per week | ( ) 5. 5–6 times per week |
| ( ) 6. Once per day | ( ) 7. Twice per day | ( ) 8. Three or more times per day |

**65. A16. How much tubers and root foods did you usually consume each time?** *[Single choice] **

| ( ) 1. 1 liang or less (≤50 g) | ( ) 2. 2 liang (100 g) | ( ) 3. 3 liang (150 g) |
| --- | --- | --- |
| ( ) 4. 4 liang (200 g) | ( ) 5. 5 liang or more (≥250 g) | ( ) 6. Did not consume |

**66. A17. How often did you consume milk, yoghurt, or milk powder during the past month?** *[Single choice] **

| ( ) 0. Never | ( ) 1. Less than once per month | ( ) 2. 1–3 times per month |
| --- | --- | --- |
| ( ) 3. 1–2 times per week | ( ) 4. 3–4 times per week | ( ) 5. 5–6 times per week |
| ( ) 6. Once per day | ( ) 7. Twice per day | ( ) 8. Three or more times per day |

**67. A18. How much milk, yoghurt, or milk powder did you usually consume each time?** *[Single choice] **

| ( ) 1. 50 mL or less | ( ) 2. 100 mL | ( ) 3. 200 mL |
| --- | --- | --- |
| ( ) 4. 300 mL | ( ) 5. 400 mL or more | ( ) 6. Did not consume |

**68. A19. How often did you consume chicken or duck eggs during the past month?** *[Single choice] **

| ( ) 0. Never | ( ) 1. Less than once per month | ( ) 2. 1–3 times per month |
| --- | --- | --- |
| ( ) 3. 1–2 times per week | ( ) 4. 3–4 times per week | ( ) 5. 5–6 times per week |
| ( ) 6. Once per day | ( ) 7. Twice per day | ( ) 8. Three or more times per day |

**69. A20. How much chicken or duck eggs did you usually consume each time?** *[Single choice] **

| ( ) 1. Half an egg or less | ( ) 2. Two-thirds of an egg | ( ) 3. 1 egg |
| --- | --- | --- |
| ( ) 4. 2 eggs | ( ) 5. 3 eggs or more | ( ) 6. Did not consume |

**70. A21. How often did you consume red-meat dishes (e.g., pork, beef, or lamb) during the past month?** *[Single choice] **

| ( ) 0. Never | ( ) 1. Less than once per month | ( ) 2. 1–3 times per month |
| --- | --- | --- |
| ( ) 3. 1–2 times per week | ( ) 4. 3–4 times per week | ( ) 5. 5–6 times per week |
| ( ) 6. Once per day | ( ) 7. Twice per day | ( ) 8. Three or more times per day |

**71. A22. How much red-meat dishes did you usually consume each time?** *[Single choice] **

Portion-size reference: 2 liang of braised pork (100 g).

| ( ) 1. 1 liang or less (≤50 g) | ( ) 2. 2 liang (100 g) | ( ) 3. 3 liang (150 g) |
| --- | --- | --- |
| ( ) 4. 4 liang (200 g) | ( ) 5. 5 liang or more (≥250 g) | ( ) 6. Did not consume |

**72. A23. How often did you consume poultry dishes (e.g., chicken, duck, or goose) during the past month?** *[Single choice] **

| ( ) 0. Never | ( ) 1. Less than once per month | ( ) 2. 1–3 times per month |
| --- | --- | --- |
| ( ) 3. 1–2 times per week | ( ) 4. 3–4 times per week | ( ) 5. 5–6 times per week |
| ( ) 6. Once per day | ( ) 7. Twice per day | ( ) 8. Three or more times per day |

**73. A24. How much poultry dishes did you usually consume each time?** *[Single choice] **

| ( ) 1. 1 liang or less (≤50 g) | ( ) 2. 2 liang (100 g) | ( ) 3. 3 liang (150 g) |
| --- | --- | --- |
| ( ) 4. 4 liang (200 g) | ( ) 5. 5 liang or more (≥250 g) | ( ) 6. Did not consume |

**74. A25. How often did you consume processed meat products (e.g., sausage, smoked meat, luncheon meat, or ham) during the past month?** *[Single choice] **

| ( ) 0. Never | ( ) 1. Less than once per month | ( ) 2. 1–3 times per month |
| --- | --- | --- |
| ( ) 3. 1–2 times per week | ( ) 4. 3–4 times per week | ( ) 5. 5–6 times per week |
| ( ) 6. Once per day | ( ) 7. Twice per day | ( ) 8. Three or more times per day |

**75. A26. How much processed meat products did you usually consume each time?** *[Single choice] **

| ( ) 1. 1 liang or less (≤50 g) | ( ) 2. 2 liang (100 g) | ( ) 3. 3 liang (150 g) |
| --- | --- | --- |
| ( ) 4. 4 liang (200 g) | ( ) 5. 5 liang or more (≥250 g) | ( ) 6. Did not consume |

**76. A27. How often did you consume freshwater fish or shellfish dishes (e.g., black carp, perch, silver carp, or river shrimp) during the past month?** *[Single choice] **

Portion-size reference from the original questionnaire: 2 liang of fish (100 g).

| ( ) 0. Never | ( ) 1. Less than once per month | ( ) 2. 1–3 times per month |
| --- | --- | --- |
| ( ) 3. 1–2 times per week | ( ) 4. 3–4 times per week | ( ) 5. 5–6 times per week |
| ( ) 6. Once per day | ( ) 7. Twice per day | ( ) 8. Three or more times per day |

**77. A28. How much freshwater fish or shellfish dishes did you usually consume each time?** *[Single choice] **

| ( ) 1. 1 liang or less (≤50 g) | ( ) 2. 2 liang (100 g) | ( ) 3. 3 liang (150 g) |
| --- | --- | --- |
| ( ) 4. 4 liang (200 g) | ( ) 5. 5 liang or more (≥250 g) | ( ) 6. Did not consume |

**78. A29. How often did you consume seafood dishes (e.g., hairtail, pomfret, yellow croaker, or sea shrimp) during the past month?** *[Single choice] **

| ( ) 0. Never | ( ) 1. Less than once per month | ( ) 2. 1–3 times per month |
| --- | --- | --- |
| ( ) 3. 1–2 times per week | ( ) 4. 3–4 times per week | ( ) 5. 5–6 times per week |
| ( ) 6. Once per day | ( ) 7. Twice per day | ( ) 8. Three or more times per day |

**79. A30. How much seafood dishes did you usually consume each time?** *[Single choice] **

| ( ) 1. 1 liang or less (≤50 g) | ( ) 2. 2 liang (100 g) | ( ) 3. 3 liang (150 g) |
| --- | --- | --- |
| ( ) 4. 4 liang (200 g) | ( ) 5. 5 liang or more (≥250 g) | ( ) 6. Did not consume |

**80. A31. How often did you consume soy-product dishes (e.g., tofu, dried tofu, vegetarian chicken, or tofu-sheet knots) during the past month?** *[Single choice] **

| ( ) 0. Never | ( ) 1. Less than once per month | ( ) 2. 1–3 times per month |
| --- | --- | --- |
| ( ) 3. 1–2 times per week | ( ) 4. 3–4 times per week | ( ) 5. 5–6 times per week |
| ( ) 6. Once per day | ( ) 7. Twice per day | ( ) 8. Three or more times per day |

**81. A32. How much soy-product dishes did you usually consume each time?** *[Single choice] **

| ( ) 1. 1 liang or less (≤50 g) | ( ) 2. 2 liang (100 g) | ( ) 3. 3 liang (150 g) |
| --- | --- | --- |
| ( ) 4. 4 liang (200 g) | ( ) 5. 5 liang or more (≥250 g) | ( ) 6. Did not consume |

**82. A33. How often did you consume nuts or seeds (e.g., watermelon seeds, sunflower seeds, walnuts, peanuts, or pistachios) during the past month?** *[Single choice] **

| ( ) 0. Never | ( ) 1. Less than once per month | ( ) 2. 1–3 times per month |
| --- | --- | --- |
| ( ) 3. 1–2 times per week | ( ) 4. 3–4 times per week | ( ) 5. 5–6 times per week |
| ( ) 6. Once per day | ( ) 7. Twice per day | ( ) 8. Three or more times per day |

**83. A34. How much nuts or seeds did you usually consume each time?** *[Single choice] **

Portion-size reference: 0.5 liang of watermelon seeds (25 g).

| ( ) 1. 1 liang or less (≤50 g) | ( ) 2. 2 liang (100 g) | ( ) 3. 3 liang (150 g) |
| --- | --- | --- |
| ( ) 4. 4 liang (200 g) | ( ) 5. 5 liang or more (≥250 g) | ( ) 6. Did not consume |

**84. A35. How often did you consume dark-coloured vegetable dishes (e.g., leafy greens, spinach, water spinach, tomatoes, green peppers, or carrots) during the past month?** *[Single choice] **

| ( ) 0. Never | ( ) 1. Less than once per month | ( ) 2. 1–3 times per month |
| --- | --- | --- |
| ( ) 3. 1–2 times per week | ( ) 4. 3–4 times per week | ( ) 5. 5–6 times per week |
| ( ) 6. Once per day | ( ) 7. Twice per day | ( ) 8. Three or more times per day |

**85. A36. How much dark-coloured vegetable dishes did you usually consume each time?** *[Single choice] **

Portion-size reference: 2 liang of leafy greens (100 g).

| ( ) 1. 1 liang or less (≤50 g) | ( ) 2. 2 liang (100 g) | ( ) 3. 3 liang (150 g) |
| --- | --- | --- |
| ( ) 4. 4 liang (200 g) | ( ) 5. 5 liang or more (≥250 g) | ( ) 6. Did not consume |

**86. A37. How often did you consume light-coloured vegetable dishes (e.g., Chinese cabbage, radish, or cucumber) during the past month?** *[Single choice] **

| ( ) 0. Never | ( ) 1. Less than once per month | ( ) 2. 1–3 times per month |
| --- | --- | --- |
| ( ) 3. 1–2 times per week | ( ) 4. 3–4 times per week | ( ) 5. 5–6 times per week |
| ( ) 6. Once per day | ( ) 7. Twice per day | ( ) 8. Three or more times per day |

**87. A38. How much light-coloured vegetable dishes did you usually consume each time?** *[Single choice] **

| ( ) 1. 1 liang or less (≤50 g) | ( ) 2. 2 liang (100 g) | ( ) 3. 3 liang (150 g) |
| --- | --- | --- |
| ( ) 4. 4 liang (200 g) | ( ) 5. 5 liang or more (≥250 g) | ( ) 6. Did not consume |

**88. A39. How often did you consume mushrooms (e.g., button mushrooms, shiitake mushrooms, straw mushrooms, or oyster mushrooms) during the past month?** *[Single choice] **

| ( ) 0. Never | ( ) 1. Less than once per month | ( ) 2. 1–3 times per month |
| --- | --- | --- |
| ( ) 3. 1–2 times per week | ( ) 4. 3–4 times per week | ( ) 5. 5–6 times per week |
| ( ) 6. Once per day | ( ) 7. Twice per day | ( ) 8. Three or more times per day |

**89. A40. How much mushrooms did you usually consume each time?** *[Single choice] **

| ( ) 1. 1 liang or less (≤50 g) | ( ) 2. 2 liang (100 g) | ( ) 3. 3 liang (150 g) |
| --- | --- | --- |
| ( ) 4. 4 liang (200 g) | ( ) 5. 5 liang or more (≥250 g) | ( ) 6. Did not consume |

**90. A41. How often did you consume fruit (e.g., apples, pears, peaches, bananas, grapes, oranges, mandarins, or watermelon) during the past month?** *[Single choice] **

| ( ) 0. Never | ( ) 1. Less than once per month | ( ) 2. 1–3 times per month |
| --- | --- | --- |
| ( ) 3. 1–2 times per week | ( ) 4. 3–4 times per week | ( ) 5. 5–6 times per week |
| ( ) 6. Once per day | ( ) 7. Twice per day | ( ) 8. Three or more times per day |

**91. A42. How much fruit did you usually consume each time?** *[Single choice] **

Portion-size reference: 3 liang of banana (150 g).

| ( ) 1. 1 liang or less (≤50 g) | ( ) 2. 2 liang (100 g) | ( ) 3. 3 liang (150 g) |
| --- | --- | --- |
| ( ) 4. 4 liang (200 g) | ( ) 5. 5 liang or more (≥250 g) | ( ) 6. Did not consume |

**92. A43. How often did you consume sugar-sweetened beverages (e.g., cola, lemon-lime soda, iced tea, or sports drinks) during the past month?** *[Single choice] **

| ( ) 0. Never | ( ) 1. Less than once per month | ( ) 2. 1–3 times per month |
| --- | --- | --- |
| ( ) 3. 1–2 times per week | ( ) 4. 3–4 times per week | ( ) 5. 5–6 times per week |
| ( ) 6. Once per day | ( ) 7. Twice per day | ( ) 8. Three or more times per day |

**93. A44. How much sugar-sweetened beverages did you usually consume each time?** *[Single choice] **

| ( ) 1. Half a cup (100 mL) | ( ) 2. 1 cup (200 mL) | ( ) 3. 1 can (350 mL) |
| --- | --- | --- |
| ( ) 4. 2 cups (400 mL) | ( ) 5. 1 bottle or more (≥550 mL) | ( ) 6. Did not consume |

**94. A45. How often did you consume beer during the past month?** *[Single choice] **

| ( ) 0. Never | ( ) 1. Less than once per month | ( ) 2. 1–3 times per month |
| --- | --- | --- |
| ( ) 3. 1–2 times per week | ( ) 4. 3–4 times per week | ( ) 5. 5–6 times per week |
| ( ) 6. Once per day | ( ) 7. Twice per day | ( ) 8. Three or more times per day |

**95. A46. How much beer did you usually consume each time?** *[Single choice] **

| ( ) 1. Half a bottle or less | ( ) 2. Two-thirds of a bottle | ( ) 3. 1 bottle (650 mL) |
| --- | --- | --- |
| ( ) 4. 2 bottles | ( ) 5. 3 bottles or more | ( ) 6. Did not consume |

**96. A47. How often did you consume yellow rice wine during the past month?** *[Single choice] **

| ( ) 0. Never | ( ) 1. Less than once per month | ( ) 2. 1–3 times per month |
| --- | --- | --- |
| ( ) 3. 1–2 times per week | ( ) 4. 3–4 times per week | ( ) 5. 5–6 times per week |
| ( ) 6. Once per day | ( ) 7. Twice per day | ( ) 8. Three or more times per day |

**97. A48. How much yellow rice wine did you usually consume each time?** *[Single choice] **

| ( ) 1. 1 liang or less (≤50 g) | ( ) 2. 2 liang (100 g) | ( ) 3. 3 liang (150 g) |
| --- | --- | --- |
| ( ) 4. 4 liang (200 g) | ( ) 5. 5 liang or more (≥250 g) | ( ) 6. Did not consume |

**98. A49. How often did you consume baijiu (Chinese distilled spirits) during the past month?** *[Single choice] **

| ( ) 0. Never | ( ) 1. Less than once per month | ( ) 2. 1–3 times per month |
| --- | --- | --- |
| ( ) 3. 1–2 times per week | ( ) 4. 3–4 times per week | ( ) 5. 5–6 times per week |
| ( ) 6. Once per day | ( ) 7. Twice per day | ( ) 8. Three or more times per day |

**99. A50. How much baijiu (Chinese distilled spirits) did you usually consume each time?** *[Single choice] **

| ( ) 1. 1 liang or less (≤50 g) | ( ) 2. 2 liang (100 g) | ( ) 3. 3 liang (150 g) |
| --- | --- | --- |
| ( ) 4. 4 liang (200 g) | ( ) 5. 5 liang or more (≥250 g) | ( ) 6. Did not consume |

# Part III. Knee Osteoarthritis Risk-Factor Screening

Section numbering retained from the original questionnaire.

**100. Have you ever had any of the following conditions?** *[Multiple choice] **

| [ ] Chronic lung disease | [ ] Coronary heart disease |
| --- | --- |
| [ ] Hypertension | [ ] Diabetes |
| [ ] Hyperlipidaemia | [ ] Hyperuricaemia |
| [ ] Cancer | [ ] Stroke |
| [ ] Other: __________ | [ ] None of the above |

**101. Family history of arthritis** *[Matrix single choice] **

| **Relative** | **Yes** | **No** | **Unknown** |
| --- | --- | --- | --- |
| Father | ○ | ○ | ○ |
| Mother | ○ | ○ | ○ |
| Full sibling(s) | ○ | ○ | ○ |
| Paternal grandfather | ○ | ○ | ○ |
| Paternal grandmother | ○ | ○ | ○ |
| Maternal grandfather | ○ | ○ | ○ |
| Maternal grandmother | ○ | ○ | ○ |

# Part IV. Educational Needs Related to Knee Osteoarthritis

**102. How willing are you to learn about the use of traditional Chinese medicine (TCM) for the treatment and prevention of knee osteoarthritis?** *[Single choice] **

| ( ) Very willing | ( ) Willing |
| --- | --- |
| ( ) Neutral | ( ) Unwilling |
| ( ) Very unwilling |  |

**103. Do you believe that external TCM treatments can effectively prevent or treat knee osteoarthritis?** *[Single choice] **

| ( ) Strongly believe | ( ) Believe |
| --- | --- |
| ( ) Neutral | ( ) Do not believe |
| ( ) Strongly disbelieve |  |

**104. Through which channels would you prefer to learn more about knee osteoarthritis?** *[Multiple choice] **

| [ ] Relevant websites | [ ] Professional books |
| --- | --- |
| [ ] Apps or official WeChat accounts | [ ] Lectures or conferences |
| [ ] Public education campaigns | [ ] Not interested in learning more |
| [ ] Other: __________ |  |

**105. Which aspects of knee osteoarthritis are you most interested in learning about?** *[Multiple choice] **

| [ ] Basic knowledge about knee osteoarthritis | [ ] Prevention of knee osteoarthritis |
| --- | --- |
| [ ] Rehabilitation knowledge and skills | [ ] Care of patients with severe knee osteoarthritis |
| [ ] Prevention and care of complications | [ ] Other: __________ |

**106. Knowledge about external TCM interventions** *[Matrix single choice] **

| **Statement** | **Strongly disagree** | **Disagree** | **Neutral** | **Agree** | **Strongly agree** |
| --- | --- | --- | --- | --- | --- |
| I know what external TCM interventions are. | ○ | ○ | ○ | ○ | ○ |
| I know the main methods of external TCM interventions. | ○ | ○ | ○ | ○ | ○ |
| I know the main indications for external TCM interventions. | ○ | ○ | ○ | ○ | ○ |
| I know the main contraindications to external TCM interventions. | ○ | ○ | ○ | ○ | ○ |
| I know the possible adverse effects of external TCM interventions. | ○ | ○ | ○ | ○ | ○ |
| I know how to find a qualified practitioner or physician who provides external TCM interventions. | ○ | ○ | ○ | ○ | ○ |
| I know how to evaluate the effectiveness of external TCM interventions. | ○ | ○ | ○ | ○ | ○ |
| I know how external TCM interventions differ from other treatments. | ○ | ○ | ○ | ○ | ○ |
| I know the costs of external TCM interventions. | ○ | ○ | ○ | ○ | ○ |
| I know the health-maintenance benefits of external TCM interventions. | ○ | ○ | ○ | ○ | ○ |

**107. Beliefs about external TCM interventions** *[Matrix single choice] **

| **Statement** | **Strongly disagree** | **Disagree** | **Neutral** | **Agree** | **Strongly agree** |
| --- | --- | --- | --- | --- | --- |
| I believe external TCM interventions are effective. | ○ | ○ | ○ | ○ | ○ |
| I believe external TCM interventions are safe. | ○ | ○ | ○ | ○ | ○ |
| I believe external TCM interventions are reliable. | ○ | ○ | ○ | ○ | ○ |
| I believe external TCM interventions are worth trying. | ○ | ○ | ○ | ○ | ○ |
| I believe external TCM interventions are acceptable to me. | ○ | ○ | ○ | ○ | ○ |
| I believe I could recommend external TCM interventions to others. | ○ | ○ | ○ | ○ | ○ |
| I believe I could adhere to external TCM interventions over the long term. | ○ | ○ | ○ | ○ | ○ |
| I believe I could perform external TCM interventions at home by myself. | ○ | ○ | ○ | ○ | ○ |
| I believe external TCM interventions can be used for disease prevention and health maintenance. | ○ | ○ | ○ | ○ | ○ |
| I believe external TCM interventions can be used in disease treatment. | ○ | ○ | ○ | ○ | ○ |

**108. Behaviours related to external TCM interventions** *[Matrix single choice] **

| **Statement** | **Strongly disagree** | **Disagree** | **Neutral** | **Agree** | **Strongly agree** |
| --- | --- | --- | --- | --- | --- |
| I have tried external TCM interventions. | ○ | ○ | ○ | ○ | ○ |
| I receive external TCM interventions regularly. | ○ | ○ | ○ | ○ | ○ |
| I perform external TCM interventions at home by myself. | ○ | ○ | ○ | ○ | ○ |
| I recommend external TCM interventions to others. | ○ | ○ | ○ | ○ | ○ |
| I use external TCM interventions for disease prevention and health maintenance. | ○ | ○ | ○ | ○ | ○ |
| I use external TCM interventions in disease treatment. | ○ | ○ | ○ | ○ | ○ |
| I ask physicians questions about external TCM interventions. | ○ | ○ | ○ | ○ | ○ |
| I learn about external TCM interventions and related knowledge. | ○ | ○ | ○ | ○ | ○ |
| I follow new developments in external TCM interventions. | ○ | ○ | ○ | ○ | ○ |
| I take part in activities related to external TCM interventions. | ○ | ○ | ○ | ○ | ○ |

**End of questionnaire**
