# Supplementary material for: Prevalence, functional impact, and associated factors of knee osteoarthritis and knee pain among adults in Urumqi, Northwest China: a community-based cross-sectional study
Source: Front Public Health. 2026 Sep 11;14:1903768. doi: 10.3389/fpubh.2026.1903768 (PMC13612206; doi:10.3389/fpubh.2026.1903768)
Supplement: Supplementary file 2 [file Data_Sheet_2.docx]

**Supplementary tables and figures**

*Data quality, subgroup burden, functional-impact models, and sensitivity analyses*

These supplementary materials summarise data quality checks, subgroup burden estimates, pain-related functional-impact models, and sensitivity analyses. Direct identifiers were not retained in any analysis output.

**Table S1: Data quality checks and construction of analytical samples.**

| Data quality item | n | Definition/handling |
| --- | --- | --- |
| Original uploaded records | 4117 | All submitted questionnaire records |
| Valid mobile phone duplicate rows removed | 116 | Duplicate defined only for valid 11-digit mobile numbers; first submission retained |
| Records after de-duplication | 4001 | Primary cleaned source |
| Adult records with valid age | 3956 | Age 18–100 years |
| Adult records with valid anthropometry and outcomes | 3880 | Height 120–220 cm; weight 30–200 kg; BMI 15–45 kg/m²; KOA and knee pain available |
| Main analysis: adults aged ≥40 years | 1925 | Primary analysis set |
| Main sample excluding duration <180 seconds | 1834 | Sensitivity analysis |
| Main sample excluding duration <300 seconds | 1345 | Sensitivity analysis |
| Likely Urumqi source within main sample | 1730 | IP or address suggested Urumqi; sensitivity analysis only |
| Questionnaires completed in <180 seconds | 136 | Flagged as very short |
| Questionnaires completed in <300 seconds | 952 | Flagged as short |
| Raw records with IP region indicating Urumqi | 3307 | Geographic quality flag |
| Raw records with address text suggesting Urumqi | 2120 | Geographic quality flag |
| Raw records with logical conflict: first diagnosis yes but ever physician diagnosis no | 74 | Sensitivity exclusion flag |
| Raw records with logical conflict: past-year pain yes but ever pain no | 115 | Sensitivity exclusion flag |
| Raw records with at least one FFQ frequency/amount inconsistency | 1656 | FFQ not used in main models |
| Raw records with straight-line KAP responses | 1699 | KAP not used in main models |

**Table S2: Burden of knee osteoarthritis and knee pain across selected subgroups.**

| Subgroup | Level | N | Physician-diagnosed KOA | Past-year knee pain | Symptomatic KOA |
| --- | --- | --- | --- | --- | --- |
| Age group | 40–49 | 621 | 11.0% (8.7–13.7) | 27.4% (24.0–31.0) | 9.7% (7.6–12.2) |
| Age group | 50–59 | 651 | 11.4% (9.2–14.0) | 27.6% (24.4–31.2) | 10.1% (8.0–12.7) |
| Age group | 60–69 | 416 | 14.2% (11.2–17.9) | 32.0% (27.7–36.6) | 12.7% (9.9–16.3) |
| Age group | ≥70 | 237 | 14.8% (10.8–19.8) | 33.3% (27.6–39.6) | 13.9% (10.1–18.9) |
| Sex | Male | 1118 | 9.3% (7.7–11.1) | 19.8% (17.5–22.2) | 8.1% (6.6–9.8) |
| Sex | Female | 807 | 16.4% (14.0–19.1) | 42.3% (38.9–45.7) | 15.1% (12.8–17.8) |
| BMI category | Underweight | 56 | 1.8% (0.3–9.4) | 16.1% (8.7–27.8) | 0.0% (0.0–6.4) |
| BMI category | Normal | 915 | 9.6% (7.9–11.7) | 26.9% (24.1–29.9) | 8.4% (6.8–10.4) |
| BMI category | Overweight | 696 | 14.9% (12.5–17.8) | 32.3% (29.0–35.9) | 13.6% (11.3–16.4) |
| BMI category | Obesity | 258 | 16.7% (12.6–21.7) | 31.8% (26.4–37.7) | 15.5% (11.6–20.4) |
| Occupational load score | 0 | 1153 | 9.5% (8.0–11.4) | 28.1% (25.6–30.8) | 8.7% (7.2–10.4) |
| Occupational load score | 1 | 239 | 15.5% (11.4–20.6) | 36.0% (30.2–42.2) | 14.2% (10.4–19.2) |
| Occupational load score | 2 | 110 | 14.5% (9.2–22.3) | 34.5% (26.3–43.8) | 13.6% (8.4–21.3) |
| Occupational load score | 3–4 | 423 | 17.3% (14.0–21.1) | 27.0% (22.9–31.4) | 14.9% (11.8–18.6) |
| Prior knee injury | No | 1741 | 8.7% (7.5–10.1) | 25.2% (23.2–27.3) | 7.9% (6.7–9.2) |
| Prior knee injury | Yes | 184 | 45.7% (38.6–52.9) | 66.8% (59.8–73.2) | 40.8% (33.9–48.0) |
| Sleep disorder | No | 1471 | 8.1% (6.8–9.6) | 21.5% (19.5–23.7) | 6.8% (5.6–8.2) |
| Sleep disorder | Yes | 454 | 25.8% (22.0–30.0) | 54.0% (49.4–58.5) | 24.7% (20.9–28.8) |
| Metabolic comorbidity count | 0 | 966 | 10.7% (8.9–12.8) | 28.5% (25.7–31.4) | 9.4% (7.7–11.4) |
| Metabolic comorbidity count | 1 | 677 | 11.8% (9.6–14.5) | 28.2% (25.0–31.7) | 10.8% (8.7–13.3) |
| Metabolic comorbidity count | ≥2 | 282 | 18.8% (14.7–23.8) | 34.0% (28.8–39.8) | 17.0% (13.1–21.8) |

Note. Data are prevalence estimates with Wilson 95% CIs.

**Table S3: Multivariable models for pain-related functional impacts among participants with past-year knee pain.**

| Predictor | Sleep impact aPR (95% CI) | Sleep impact P | Work/activity impact aPR (95% CI) | Work/activity impact P | Medical help seeking aPR (95% CI) | Medical help seeking P | Any sleep or work/activity impact aPR (95% CI) | Any sleep or work/activity impact P |
| --- | --- | --- | --- | --- | --- | --- | --- | --- |
| Age (per 10 years) | 0.99 (0.89–1.11) | 0.890 | 1.00 (0.92–1.08) | 0.976 | 1.04 (0.97–1.12) | 0.240 | 0.99 (0.92–1.07) | 0.838 |
| Female sex | 1.08 (0.85–1.38) | 0.520 | 0.90 (0.75–1.08) | 0.252 | 0.94 (0.80–1.10) | 0.425 | 0.95 (0.79–1.13) | 0.543 |
| First-degree family history of arthritis | 1.00 (0.81–1.23) | 0.980 | 0.99 (0.85–1.17) | 0.949 | 1.08 (0.94–1.24) | 0.279 | 1.04 (0.90–1.21) | 0.576 |
| Obesity | 1.26 (0.94–1.70) | 0.117 | 1.45 (1.15–1.83) | 0.002 | 1.27 (1.07–1.52) | 0.008 | 1.37 (1.10–1.70) | 0.005 |
| Occupational load score 3–4 | 1.42 (1.08–1.85) | 0.011 | 1.44 (1.18–1.75) | <0.001 | 1.21 (1.02–1.43) | 0.025 | 1.41 (1.17–1.71) | <0.001 |
| Overweight | 1.22 (0.98–1.52) | 0.073 | 1.40 (1.19–1.66) | <0.001 | 1.17 (1.01–1.36) | 0.042 | 1.37 (1.17–1.61) | <0.001 |
| Prior knee injury | 1.57 (1.27–1.93) | <0.001 | 1.30 (1.11–1.52) | 0.001 | 1.39 (1.22–1.58) | <0.001 | 1.28 (1.10–1.48) | 0.001 |
| Sleep disorder | 1.43 (1.17–1.76) | <0.001 | 1.22 (1.05–1.42) | 0.011 | 1.09 (0.95–1.25) | 0.199 | 1.33 (1.15–1.54) | <0.001 |
| ≥2 metabolic comorbidities | 1.46 (1.12–1.89) | 0.004 | 1.44 (1.17–1.78) | <0.001 | 1.24 (1.04–1.48) | 0.017 | 1.35 (1.11–1.65) | 0.003 |

Note. Models were fitted among participants reporting knee pain in the past 12 months. Estimates are aPRs with 95% CIs.

**Table S4: Sensitivity analyses for selected correlates.**

| Analysis set | Outcome | N | Events | Obesity | Sleep disorder | Occupational load score 3–4 | Prior knee injury |
| --- | --- | --- | --- | --- | --- | --- | --- |
| Primary main sample (≥40 years) | Physician-diagnosed KOA | 1925 | 236 | 1.39 (1.00–1.92) | 2.04 (1.59–2.62) | 1.48 (1.09–2.03) | 3.08 (2.37–4.00) |
| Primary main sample (≥40 years) | Past-year knee pain | 1925 | 562 | 1.06 (0.87–1.29) | 1.81 (1.58–2.08) | 0.93 (0.78–1.12) | 2.01 (1.72–2.34) |
| Excluding duration <180 s | Physician-diagnosed KOA | 1834 | 232 | 1.36 (0.97–1.89) | 1.97 (1.54–2.53) | 1.52 (1.11–2.09) | 3.02 (2.33–3.92) |
| Excluding duration <180 s | Past-year knee pain | 1834 | 553 | 1.07 (0.88–1.30) | 1.78 (1.55–2.04) | 0.95 (0.80–1.14) | 1.98 (1.70–2.30) |
| Excluding duration <300 s | Physician-diagnosed KOA | 1345 | 193 | 1.33 (0.92–1.91) | 1.73 (1.33–2.25) | 1.74 (1.24–2.45) | 2.50 (1.90–3.29) |
| Excluding duration <300 s | Past-year knee pain | 1345 | 469 | 1.06 (0.86–1.30) | 1.57 (1.36–1.81) | 1.05 (0.87–1.26) | 1.83 (1.57–2.14) |
| Likely Urumqi by IP/address | Physician-diagnosed KOA | 1730 | 202 | 1.32 (0.92–1.88) | 1.75 (1.33–2.30) | 1.56 (1.11–2.20) | 3.21 (2.42–4.25) |
| Likely Urumqi by IP/address | Past-year knee pain | 1730 | 511 | 1.04 (0.84–1.28) | 1.71 (1.48–1.98) | 0.89 (0.73–1.08) | 1.93 (1.64–2.28) |
| Excluding logical conflict records | Physician-diagnosed KOA | 1862 | 227 | 1.47 (1.05–2.04) | 2.08 (1.62–2.68) | 1.57 (1.14–2.15) | 3.16 (2.43–4.11) |
| Excluding logical conflict records | Past-year knee pain | 1862 | 514 | 0.99 (0.80–1.23) | 1.90 (1.64–2.20) | 0.96 (0.79–1.16) | 2.06 (1.75–2.43) |
| Adult sample (≥18 years) | Physician-diagnosed KOA | 3880 | 378 | 1.31 (1.03–1.68) | 2.05 (1.68–2.50) | 1.24 (0.97–1.57) | 4.25 (3.47–5.21) |
| Adult sample (≥18 years) | Past-year knee pain | 3880 | 1000 | 1.12 (0.96–1.30) | 1.59 (1.43–1.77) | 1.04 (0.92–1.19) | 2.53 (2.27–2.82) |

Note. Each row reports aPRs (95% CIs) for selected factors using the same covariate structure as the primary models.

**Figure S1: Past-year knee pain prevalence by age group and sex.**

**Figure S2: BMI distribution by self-reported physician-diagnosed knee osteoarthritis.**
